# A CCR5 antagonist, maraviroc, alleviates neural circuit dysfunction and behavioral disorders induced by prenatal valproate exposure

**DOI:** 10.1186/s12974-022-02559-y

**Published:** 2022-07-29

**Authors:** Yasuhiro Ishihara, Tatsuya Honda, Nami Ishihara, Kaede Namba, Makiko Taketoshi, Yoko Tominaga, Mayumi Tsuji, Christoph F. A. Vogel, Takeshi Yamazaki, Kouichi Itoh, Takashi Tominaga

**Affiliations:** 1 Program of Biomedical Science, Graduate School of Integrated Sciences for Life, Hiroshima University, 1-7-1, Kagamiyama, Higashi-Hiroshima, Hiroshima, 739-8521 Japan; 2 Center for Health and the Environment, University of California, Davis, Davis, CA 95616 USA; 3 Institute of Neuroscience, Tokushima Bunri University, Kagawa, 769-2193 Japan; 4 Department of Environmental Health, University of Occupational and Environmental Health, Fukuoka, 807-8555 Japan; 5 Department of Environmental Toxicology, University of California, Davis, Davis, CA 95616 USA; 6 Program of Life and Environmental Sciences, Graduate School of Integrated Sciences for Life, Hiroshima University, Hiroshima, 739-8521 Japan; 7 Laboratory for Pharmacotherapy and Experimental Neurology, Kagawa School of Pharmaceutical Sciences, Tokushima Bunri University, Kagawa, 769-2193 Japan

**Keywords:** Valproic acid, Microglia, Neuroinflammation, CCL3, Neural circuit, Behavioral disorders, Maraviroc

## Abstract

**Background:**

Valproic acid (VPA) is a clinically used antiepileptic drug, but it is associated with a significant risk of a low verbal intelligence quotient (IQ) score, attention-deficit hyperactivity disorder and autism spectrum disorder in children when it is administered during pregnancy. Prenatal VPA exposure has been reported to affect neurogenesis and neuronal migration and differentiation. In addition, growing evidence has shown that microglia and brain immune cells are activated by VPA treatment. However, the role of VPA-activated microglia remains unclear.

**Methods:**

Pregnant female mice received sodium valproate on E11.5. A microglial activation inhibitor, minocycline or a CCR5 antagonist, maraviroc was dissolved in drinking water and administered to dams from P1 to P21. Measurement of microglial activity, evaluation of neural circuit function and expression analysis were performed on P10. Behavioral tests were performed in the order of open field test, Y-maze test, social affiliation test and marble burying test from the age of 6 weeks.

**Results:**

Prenatal exposure of mice to VPA induced microglial activation and neural circuit dysfunction in the CA1 region of the hippocampus during the early postnatal periods and post-developmental defects in working memory and social interaction and repetitive behaviors. Minocycline, a microglial activation inhibitor, clearly suppressed the above effects, suggesting that microglia elicit neural dysfunction and behavioral disorders. Next-generation sequencing analysis revealed that the expression of a chemokine, C–C motif chemokine ligand 3 (CCL3), was upregulated in the hippocampi of VPA-treated mice. CCL3 expression increased in microglia during the early postnatal periods via an epigenetic mechanism. The CCR5 antagonist maraviroc significantly suppressed neural circuit dysfunction and post-developmental behavioral disorders induced by prenatal VPA exposure.

**Conclusion:**

These findings suggest that microglial CCL3 might act during development to contribute to VPA-induced post-developmental behavioral abnormalities. CCR5-targeting compounds such as maraviroc might alleviate behavioral disorders when administered early.

**Supplementary Information:**

The online version contains supplementary material available at 10.1186/s12974-022-02559-y.

## Introduction

Valproic acid (VPA), a broad-spectrum antiepileptic drug, has been widely used to treat almost all types of seizures and epilepsy syndromes, although its greatest value is in the management of generalized epilepsies associated with multiple seizure types. The actions of VPA that have been reported thus far include potentiation of gamma-aminobutyric acid (GABA) transmission through increased GABA synthesis, decreased GABA turnover, and inhibition of GABA degradation; inhibition of NMDA receptor-mediated excitatory transmission; blockade of voltage-gated sodium channels; and blockade of calcium channels, which can explain the broad-spectrum anti-seizure effects of VPA [1]. However, VPA is known to have several adverse effects, especially when it is administered during pregnancy. School-age children whose mothers had been given VPA during pregnancy had significantly lower verbal intelligence quotient scores than children exposed to carbamazepine or phenytoin or children not exposed to antiepileptic drugs [2]. Prenatal exposure to VPA also reportedly increases the risk for attention-deficit hyperactivity disorder [3] and autism spectrum disorder [4]. Based on these findings, restriction of VPA administration to pregnant women and women of childbearing potential is recommended in many countries. The risk–benefit balance of VPA treatment in women should be deeply considered [5].

The mechanisms of behavioral defects induced by prenatal VPA exposure have been investigated using animal models. VPA has been reported to decrease the expression of the NMDAR subunits NR2A and NR2B in the primary somatosensory cortex to enhance NMDA receptor-mediated synaptic plasticity in rats [6]. VPA disrupts the normal excitatory–inhibitory shift of GABAergic currents during postnatal development [7]. Mice exposed to VPA in utero exhibit impaired cognitive function as adults, and this effect is related to decreased hippocampal neurogenesis [8]. The ectopic localization of newborn neurons in the hippocampus increases seizure susceptibility in adult mice prenatally exposed to VPA [9]. Recently, the effects of prenatal VPA exposure on astrocytes in addition to neurons have been studied. VPA activates astrocytes to alter the expression of the glutamate transporter GLT-1/EAAT-2, which disturbs glutamate metabolism [10]. Thus, several mechanisms may be involved in fetal VPA exposure-induced post-developmental behavioral disorders. However, the action of VPA on neurons has been studied, and its effects on immune cells remain unclear.

Microglia are immune cells in the central nervous system that play important roles in brain pathologies such as Alzheimer's disease and epilepsy [11, 12]. Microglia are also involved in neuronal circuit formation during development. Microglia engulf presynaptic inputs during peak retinogeniculate pruning. Engulfment is dependent upon neural activity and the microglia-specific complement receptor 3 phagocytic signaling pathway [13]. Increasing evidence has shown that microglia may be targets of VPA in the developmental stage. Prenatal VPA administration upregulates CD11b expression in the hippocampus [14] and increases the number of microglia in the hippocampal CA1 region [15]. Zamberietti et al. reported that fetal VPA treatment induces an increase in the soma area of microglia in the hippocampus [16]. The phagocytic activity of BV-2 mouse microglial cells against amyloid beta is enhanced in the presence of VPA [17]. Based on these findings and the importance of microglia in development, elucidating the effect of VPA on microglia might be important for understanding the mechanism of abnormal behaviors induced by prenatal VPA exposure. The purpose of this study was to examine the effects of VPA on microglia to identify a target for treating post-developmental behavioral disorders, focusing on neuron–microglia interactions during development.

VPA has been used for making an autism model and thus there are several administration protocols. The timing of administration as well as the dosage of VPA determine the effects. Mice exposed to VPA on embryonic day 12.5 (E12.5) but not on E9 and E14.5 have been shown to exhibit autistic-like behavioral alternations [18]. Similarly, rats exposed to VPA on E12.5 but not on E7, 9.5 and 15 display the changes in social behaviors [19]. In human, VPA exposure during the first trimester of pregnancy can induce neural tube defects and other congenital malformations such as atrial septal defect, cleft palate, hypospadias, polydactyly and craniosynostosis [20–22]. Administration of VPA on days 8–9 of gestation in mice results in failure of cranial neural tube closure and spina bifida, as well as limb abnormalities such as syndactyly and oligodactyly [23]. Thus, the pathophysiology of mice is similar to that of human. Treatment with VPA during midgestation can induce post-developmental behavioral disorders and oral administration at the dosage of 800 mg/kg VPA was widely used when exposed on E11 [24, 25]. Therefore, we selected this protocol in our study.

## Materials and methods

### Animals and drug administration

**Fig. 1:**
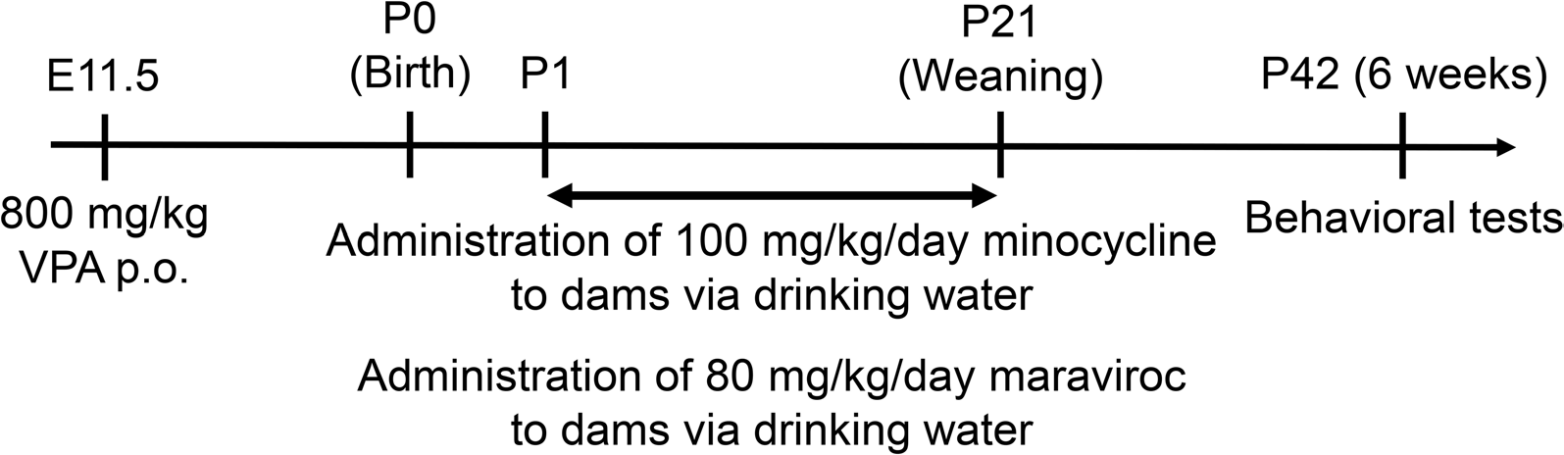
Experimental protocol. Pregnant female mice received a single oral dose of 800 mg/kg sodium valproate on E11.5. Minocycline (100 mg/kg/day) or maraviroc (80 mg/kg/day) was dissolved in drinking water and administered to dams from P1 to P21. Behavioral tests were performed in the order of open field test, Y-maze test, social affiliation test and marble burying test from the age of 6 weeks

All animal procedures were performed in accordance with the Fundamental Guidelines for Proper Conduct of Animal Experiments and Related Activities in Academic Research Institutions under the Jurisdiction of the Ministry of Education, Culture, Sports, Science and Technology, Japan. The Animal Care and Use Committee of Hiroshima University approved the experimental protocols (No. C18-23-4). Pregnant ICR mice were obtained from Japan SLC (Shizuoka, Japan) and were maintained in a temperature-controlled animal facility on a 12-h light–dark cycle (light on from 8:00 a.m. to 8:00 p.m.). Mice were allowed ad libitum access to diet and water. Sodium valproate (Sigma-Aldrich, St. Louis, MO, USA) was dissolved in saline. Pregnant female mice received a single dose of 800 mg/kg sodium valproate by gavage on day 11.5 after conception. Minocycline (Wako Pure Chemical, Osaka, Japan; 100 mg/kg/day) was dissolved in drinking water at the required concentration, which was determined based on each dam's body weight and drinking volume, and given to the dams from 1 day old (P1) to 21 days old (P21) via lactation. Maraviroc (GlaxoSmithKline, London, UK; 80 mg/kg/day) was dissolved in drinking water, sonicated, warmed and administered to the dams from P1 to P21 via lactation. The administration schedule is shown in Fig. 1. We used simple randomization for the randomization method [26]. Four to five mice were housed in the same cage from P22. The brain tissues were perfused with phosphate-buffered saline under isoflurane anesthesia and then isolated. No sample calculation was performed and that sample size was determined by our previous studies and other reports [25, 27–29]. Any animal was not excluded based on the statistics or died during experiments. One hundred thirty-two dams and 322 male pups were used in this study. *N* values and details of litters are included in figure legends as appropriate.

### Behavioral tests

Eye opening was sequentially observed after birth and scored as 0.5 for one eye open and 1 for both eyes open to evaluate development. Behavioral tests were performed by well-trained investigators who were blinded to the experimental groups. Data analyses were also performed by technical staff independently. Behavioral tests were performed in the order of open field test, Y-maze test, social affiliation test and marble burying test from the age of 6 weeks. All behavioral tests were performed from 8:00 am to 12:00 am. In the open field test, the mice were released into an open field arena (90 × 90 × 50 cm). The arena was equipped with a camera mounted overhead. The mice were monitored for 90 min, and the distance moved was calculated by DIPP-Motion V/2D software (DITECT, Tokyo, Japan).

The Y-maze test was performed using an apparatus with three arms (20 cm long × 10 cm wide × 20 cm high) at 120° angles. The mice were placed in the distal end of an arm and allowed to explore the maze for 8 min. The percentage of alternations (entries into an arm that differed from the arm entered in the previous two entries) was calculated with the following formula: (alternations/(arms entries – 2)) × 100 [30].

A social affiliation test was performed in a rectangular apparatus (20 cm long × 40 cm wide × 40 cm high). Empty wire containment cups were placed on the right and left sides 8 cm from the wall, and the mice were habituated to the apparatus for 5 min. A control mouse (stranger) was placed in a wire containment cup that was located on one of the sides of the apparatus, and then the mice were placed in the apparatus for 10 min. A video camera mounted above the apparatus recorded the movements of the mice for analysis. The amount of time spent around each cage (empty cage or stranger-containing cage) was measured using DIPP-Motion V/2D software [31].

The marble burying test was performed in a mouse cage (22 cm long × 32 cm wide × 15 cm high), in which 20 glass marbles (diameter 1.2 cm) were spaced evenly on a 2-cm-deep layer of shaved wood bedding. The mice were placed in the cage and left undisturbed for 30 min. After the test, the number of marbles buried (covered more than two-thirds with bedding) was counted [32].

### Immunohistochemistry (IHC)

IHC was performed according to our previous report [33]. Briefly, the brains were fixed with 4% buffered paraformaldehyde and cryoprotected in 30% sucrose. Brains were frozen, and 50-μm-thick floating sections were prepared using a Cryostat (CM3050 S; Leica Biosystems, Nussloch, Germany). The sections were blocked and permeabilized with PBS containing 10% normal goat serum (Sigma-Aldrich) and 0.3% Triton-X 100 for 1 h at room temperature. The sections were incubated with primary antibody for 3 h at room temperature, followed by secondary antibody for 1 h at room temperature in the dark. The antibodies used are listed in Additional file 1: Table S1. The sections were mounted on a glass slide with DAPI-Fluoromount-G (Southern Biotech, Birmingham, AL, USA). Images were processed using the Zen image acquisition software package (Carl Zeiss, Oberkochen, Germany) and ImageJ software. The soma area was evaluated using images of Iba1 staining, and the amoeboid score was calculated by the following formula: (soma area/entire Iba1-stained area) × 100 (%). The Iba1- and CD68-costained areas were analyzed with ImageJ software (National Institutes of Health, Bethesda, MD, USA). At least 100 cells were measured for calculating the amoeboid score and CD68-stained area.

### Analysis of minocycline and maraviroc levels by HPLC–UV

Minocycline levels in the serum and brain were measured as described in a previous report with slight modifications [34]. Hippocampal homogenates were prepared in phosphate-buffered saline. Fifty microlitres of each homogenate was mixed with 20 μL of 0.5 M dipotassium hydrogen phosphate aqueous solution and 250 μL of ethyl acetate. The mixture was vortexed and centrifuged, and the resulting upper layer (ethyl acetate layer) was isolated. Fifty microlitres of 20 mM hydrochloric acid was added to the ethyl acetate solution. The mixture was vortexed and centrifuged, and 20 μL of the lower layer (water layer) was injected onto an InertSustain Cyano column (4.0 × 250 mm, 5 µm) (GL Science, Tokyo, Japan). The mobile phase consisted of methanol and 20 mM perchloric acid/4 mM triethylamine in water (20:80, v/v; pH approx. 2). The flow rate was 1 mL/min, the column temperature was 25 °C, and UV detection was performed at 350 nm. Solutions of minocycline (Wako Pure Chemical) were used as standards.

The maraviroc concentration was determined as described in a previous report with slight modifications [35]. Equal amounts of serum or hippocampal homogenates were mixed with acetonitrile. After the mixtures were vortexed and centrifuged, 50 μL of supernatant was injected onto an InertSustain Cyano column (4.0 × 250 mm, 5 µm) (GL Science). The mobile phase consisted of acetonitrile and 20 mM potassium dihydrogenphosphate in water, and the pH was adjusted to 2.5 by phosphoric acid (40:60, v/v). The flow rate was 0.6 mL/min, the column temperature was 25 °C, and UV detection was performed at 210 nm. Solutions of known concentrations of maraviroc (Cayman Chemical Company, Ann Arbor, MI, USA) were used as standards.

### Voltage-sensitive dye (VSD) imaging

VSD imaging was performed using the same techniques as those described in previous reports [36, 37]. Briefly, hippocampal slices (350 µm) were prepared and transferred onto a fine-mesh membrane filter (Omni Pore membrane filter, JHWP01300; Millipore) held in place by a thin Plexiglas ring (inner diameter, 11 mm; outer diameter, 15 mm; thickness, 1–2 mm). The slices were stained for 25 min with 100 µL of VSD solution [0.2 mM di-4-ANEPPS (Thermo Fisher Scientific, Waltham, MA, USA) in 2.5% ethanol, 0.13% Cremaphor EL (Sigma-Aldrich), 1.17% distilled water, 48.1% FBS, and 48.1% artificial cerebrospinal fluid (ACSF)]. The slices were used for experiments after at least a 1-h incubation at room temperature following VSD washout.

The Plexiglas ring supporting each slice (see above) was placed in an immersion-type recording chamber [38]. Custom laboratory-designed epifluorescence optics consisting of a custom-made objective lens (Brainvision 6×, water immersion) and a Leica Microsystems MZ-APO (*f* = 55 mm × 1.0) projection lens were used to view the slices during the experiments. Excitation light was provided by a high-power stabilized LED light (LEX-2G; Brainvision Co Ltd, Tokyo, Japan) projected through an excitation filter (λ = 530 ± 10 nm) and reflected onto the hippocampal slice by a dichroic mirror (*λ* = 575 nm). Emission fluorescence from the slice passed through an emission filter (*λ* > 590 nm) and was projected onto a C-MOS imager (MiCAM-02; Brainvision). The intensity of fluorescence emitted by the slice prior to stimulation was averaged and used as the reference intensity (*F*0). The fractional change in fluorescence [*ΔF*(*t*) = *F*(*t*)-*F*0] was normalized to *F*0 (*ΔF*/*F*0), and this value was used as the optical signal. The optical signals referred to below represent signals filtered in spatial and temporal dimensions with a digital Gaussian kernel of 5 × 5 × 3 (horizontal × vertical × temporal; *σ* ≈ 1). At a wavelength of 610 nm, VSD fluorescence decreased in response to depolarization of the cell membrane. Electrical stimulations (40 V, bipolar 200 µs) were applied with constant voltage pulses (ESTM-8, Brainvision Co. Ltd.) through a glass microcapillary tube (5 µm inner diameter; filled with ACSF) placed either on Schaffer collateral afferents in the CA3/CA1 border of CA1, on the granule cell layer to stimulate the mossy fiber pathway, or in the molecular layer of the upper blade of the dentate gyrus (DG). Stimulation was applied at an interval of at least 30 s.

### Cap analysis gene expression (CAGE)-seq analysis

RNA was prepared from the hippocampi of 5-day-old mice exposed to vehicle or VPA using a ReliaPrep RNA Miniprep System (Promega, Madison, WI, USA). RNA isolated from 5 male pups from 2 dams in each group was analyzed by CAGE-seq. RNA quality was assessed with a Bioanalyzer (Agilent, Santa Clara, CA, USA) to ensure that the RNA integrity number (RIN) was over 8.0 and that the A260/A280 and 260/230 ratios were over 2.0. CAGE library preparation, sequencing, mapping, and gene expression analysis were performed by DNAFORM (Yokohama, Kanagawa, Japan). First-strand cDNA was transcribed to the 5′ end of capped RNAs and attached to CAGE barcode tags, and these tags were sequenced using the NextSeq 500 system (Illumina, San Diego, CA, USA) and mapped to the mouse mm9 genomes using the BWA software program after discarding ribosomal RNAs. Over 20 million reads were mapped to the murine genome sequence for each sample. The data were analyzed, and the expression ratio was also calculated as the log (base 2) ratio using the RECLU pipeline. Raw data were registered in the NCBI GEO database (accession no. GSE180564).

### Total RNA extraction and quantitative PCR (qPCR)

mRNA levels were determined according to the protocol described in our previous report [39]. Briefly, total RNA was extracted from the hippocampus using a High Pure RNA Isolation Kit (Roche Diagnostics K.K., Tokyo, Japan). Single-stranded cDNA was synthesized from 1 μg of total RNA according to the ReverTra Ace protocol (Toyobo, Osaka, Japan) with a random primer (9-mer; Takara Bio, Shiga, Japan). Real-time PCR was performed using a CFX Connect real-time PCR system (Bio–Rad Laboratories, Hercules, CA, USA) with TB Green Premix Ex Ta II (TaKaRa Bio, Shiga, Japan). The primer sequences are presented in Additional file 1: Table S2. mRNA levels were normalized to the level of the housekeeping gene β-actin, and the values of the treated samples were divided by those of the untreated samples to calculate the relative mRNA levels.

### ELISA for C–C motif chemokine ligand 3 (CCL3)

Freshly isolated hippocampi were lysed in radioimmunoprecipitation assay (RIPA) buffer containing cOmplete EDTA-free Protease Inhibitor Cocktail (Roche). The hippocampal CCL3 concentration was determined using the Mouse CCL3/MIP-1 Alpha Quantikine ELISA Kit (R&D Systems, Inc., Minneapolis, MN, USA) according to the manufacturers’ instructions.

### Immunoblotting

CD11b-positive cells were isolated according to our previously described method [40]. Cells or tissues were lysed with RIPA buffer (25 mM Tris–HCl (pH 7.6), 150 mM NaCl, 1% Nonidet P-40, 1% sodium deoxycholate, and 0.1% SDS). Equal amounts of protein were loaded, separated via SDS-PAGE and transferred onto polyvinylidene difluoride membranes. The blocked membranes were incubated with the primary and secondary antibodies listed in Additional file 1: Table S1. Then, the membranes were visualized using peroxide substrates (SuperSignal West Dura, Thermo Fisher Scientific).

### Statistical analyses

**Fig. 2:**
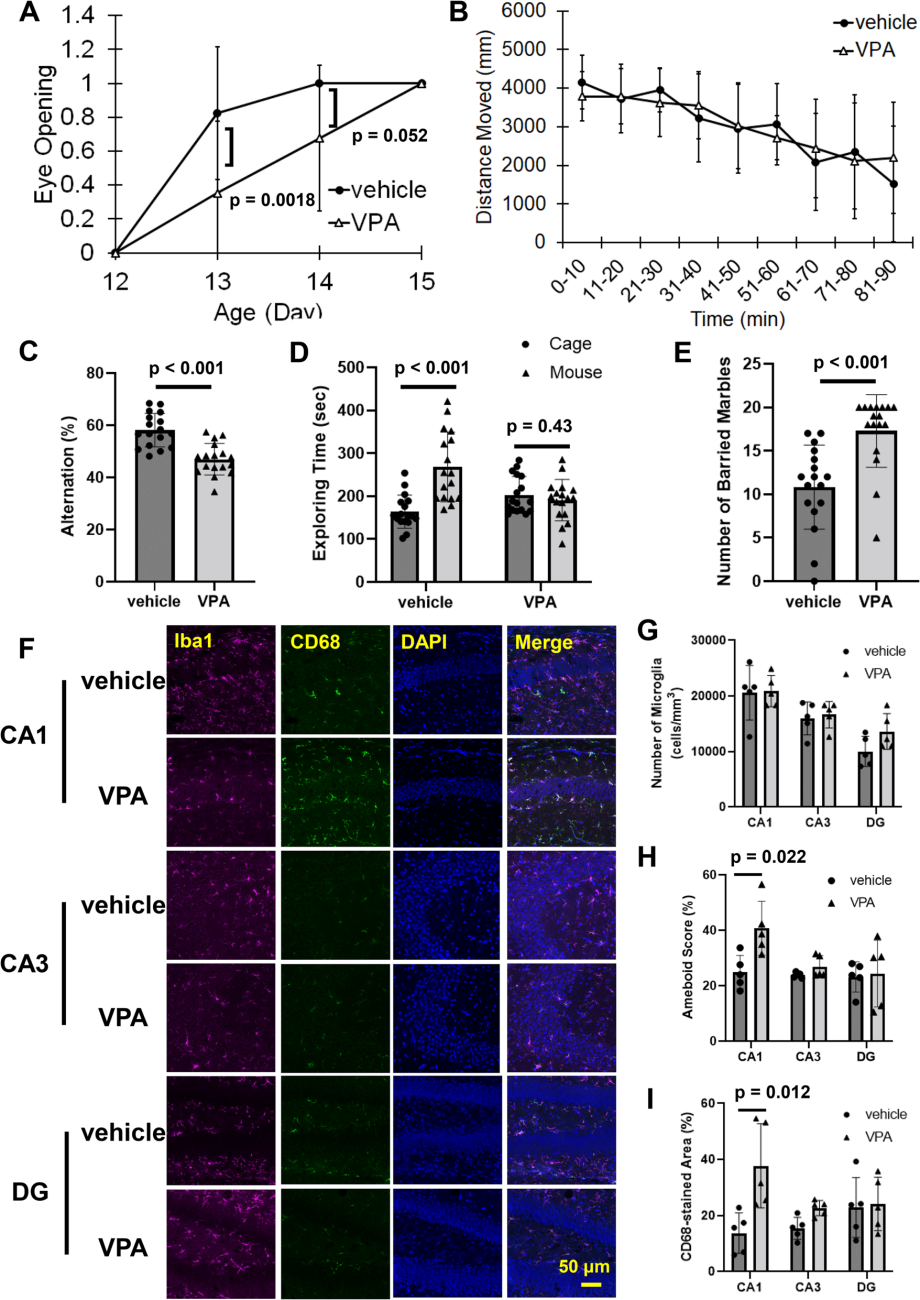
Microglial activation during development and post-developmental behavioral disorders induced by prenatal exposure to VPA. VPA (800 mg/kg) was orally administered on E11.5. **A** Eye opening was sequentially observed and scored as 0.5 for one eye open and 1 for both eyes open. The values are presented as the mean ± S.D. (*n* = 17 male pups from 3 dams in each group). The data were analyzed using two-way ANOVA [*F*(1, 64) = 0.7067, *p* = 0.4037] with Tukey's corrected multiple comparison tests. **B** Locomotor activity evaluated by the open field test at the age of 6 weeks. The values are presented as the mean ± S.D. (*n* = 17 male pups from 3 dams in each group). The data were analyzed using two-way ANOVA [*F*(8, 288) = 1.012, *p* = 0.4273] with Tukey's corrected multiple comparison tests. **C**–**E** The number of alternations, exploration time and the number of buried marbles were measured in the *Y*-maze, social affiliation and marble burying tests, respectively. The values are presented as the mean ± S.D. (*n* = 17 male pups from 3 dams in each group). The data were analyzed using Student's *t* test. **F**–**I** Hippocampal slices were prepared from P10 mice and stained for Iba1, CD68 and DAPI. **F** Representative stained pictures. **G** The number of microglia in the CA1, CA3 and DG regions. **H** The amoeboid score was calculated from Iba1 staining images. **I** The CD68-stained area was determined by calculating the Iba1/CD68 staining images. The values are presented as the mean ± S.D. (*n* = 5 male pups from 2 dams in each group). The data were analyzed using Student's *t* test

**Fig. 3:**
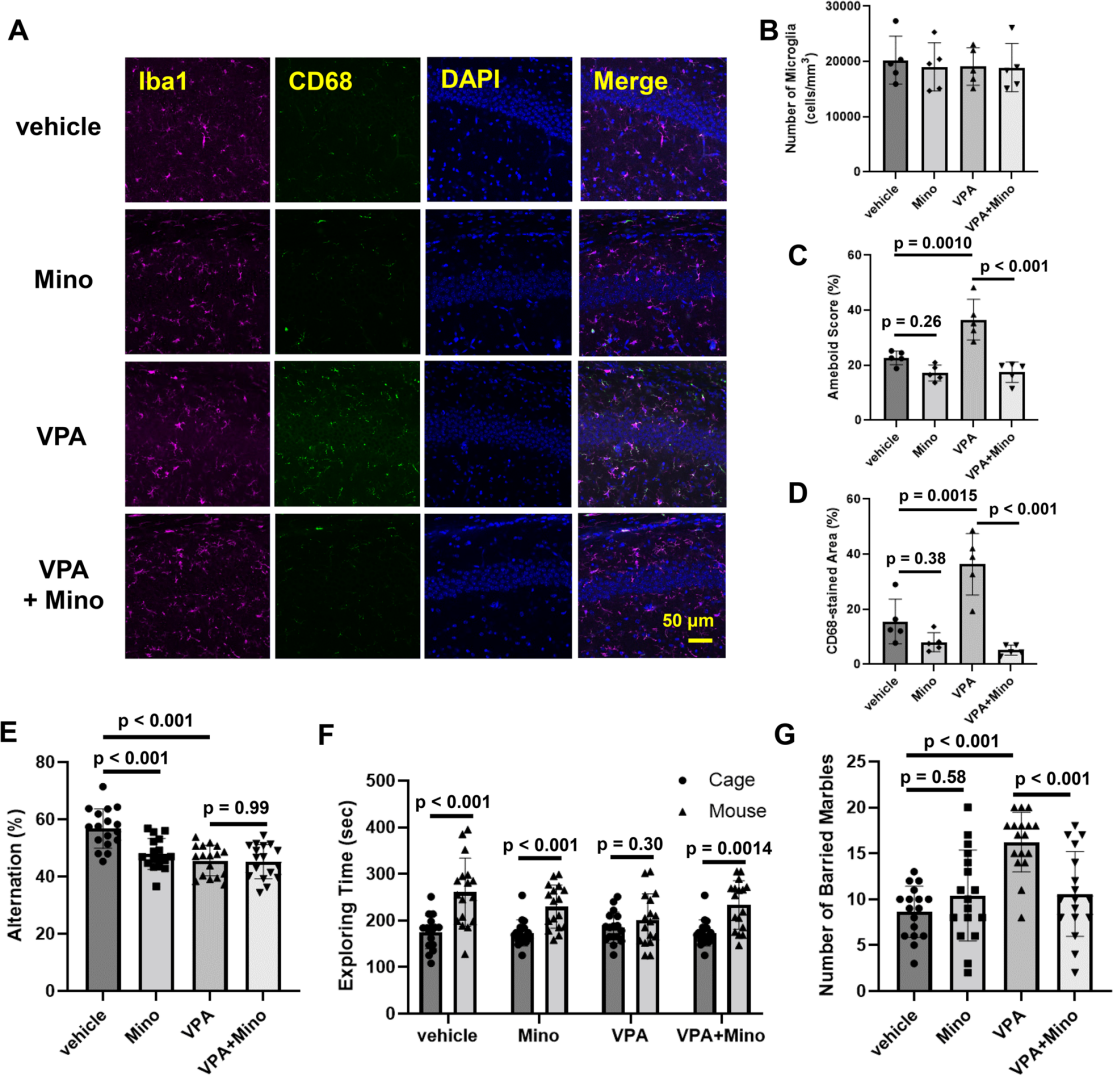
Involvement of activated microglia in VPA-mediated induction of behavioral disorders. VPA (800 mg/kg) was orally administered on E11.5. Minocycline (100 mg/kg) was administered to dams by drinking water from P1 to P21. **A**–**D** Hippocampal slices obtained from P10 mice were stained with Iba1 and CD68 and observed by confocal microscopy. **A** Representative stained pictures. **B** The number of microglia. **C** Amoeboid scores and **D** the CD68-stained area. The values are presented as the mean ± S.D. (*n* = 5 male pups from 2 dams in each group). The data were analyzed using two-way ANOVA [**B** *F*(1, 16) = 0.07785, *p* = 0.7838, **C** *F*(1, 16) = 11.11, *p* = 0.0042, **D** *F*(1, 16) = 13.67, *p* = 0.0020] with Tukey's corrected multiple comparison tests. **E** The number of alternations in the Y-maze test. The values are presented as the mean ± S.D. (*n* = 17 male pups from 3 dams in each group). The data were analyzed using two-way ANOVA [*F*(1, 64) = 8.723, *p* = 0.0044] followed by Tukey's *t* test. **F** Social interaction, as measured by exploration time in the open field. The values are presented as the mean ± S.D. (*n* = 17 male pups from 3 dams in each group). The data were analyzed using Student's *t* test. **G** The number of buried marbles. The values are presented as the mean ± S.D. (*n* = 17 male pups from 3 dams in each group). The data were analyzed using two-way ANOVA [*F*(1, 64) = 14.55, *p* = 0.0003] followed by Tukey's *t *test

**Fig. 4:**
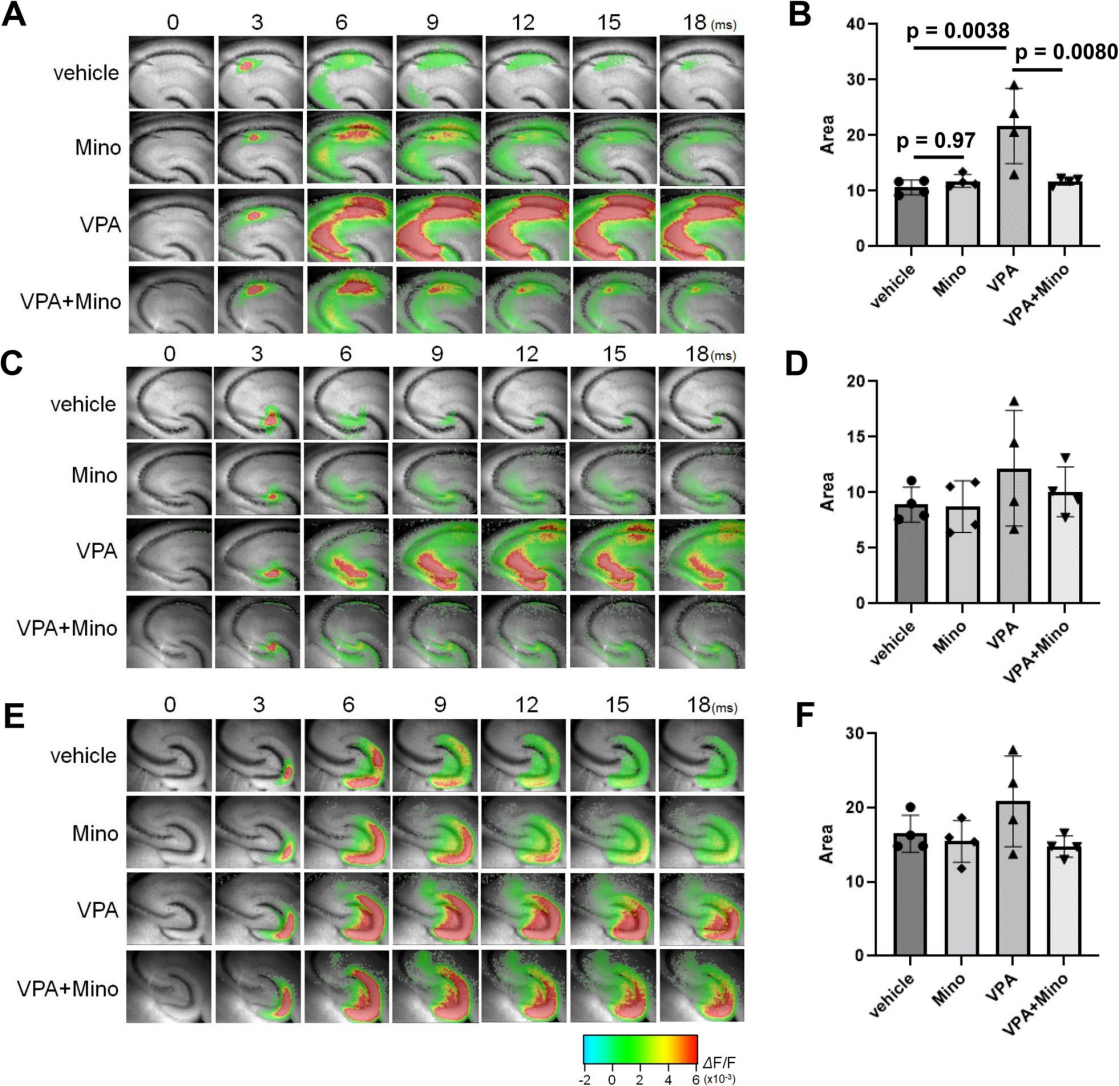
Involvement of microglia in the induction of a neuronal excitation–inhibition imbalance by VPA. Pregnant female mice received a single oral dose of 800 mg/kg sodium valproate on E11.5. Minocycline (100 mg/kg) was administered to dams by drinking water from P1 to P10. Hippocampal slices were prepared from 10-day-old male pups, and then electrical stimulation was applied to Schaffer collateral afferents at the CA3/CA1 border in the CA1 region (**A**, **B**), the granule cell layer to stimulate the mossy fiber pathway (**C**, **D**), and the molecular layer of the upper blade of the DG (**E**, **F**) in hippocampal slices from P10 mice. **A**, **C**, **E** Representative pseudocolored images showing activity. **B**, **D**, **F** Quantification of the neural response in the slices. The values are presented as the mean ± S.D. (*n* = 4 male pups from 2 dams in each group). The values are presented as the mean ± S.D. (*n* = 5 male pups from 2 dams in each group). The data were analyzed using two-way ANOVA [**B** *F*(1, 12) = 10.04, *p* = 0.0081, **D** *F*(1, 12) = 5.021, *p* = 0.0447, **F** *F*(1, 12) = 1.910, *p* = 0.1921] with Tukey's corrected multiple comparison tests

All data were analyzed using GraphPad Prism 9 (GraphPad Software, San Diego, CA, USA). Student's *t* test, one-way ANOVA with Dunnett's corrected multiple comparison tests, two-way ANOVA [VPA × Age (Fig. 2A), VPA × time points (Fig. 2B), VPA × Mino (Figs. 3B–E, G, 4B, D, F and 6A, B) or VPA × Mar (Fig. 7B–E, G)] with Tukey's corrected multiple comparison tests were used to determine significant differences between the means of two or more independent groups of animals. The *F* value calculated from ANOVA is described in each figure legend, the p value is indicated in each figure, and significance was considered when *p* values were less than 0.05. Error bars were calculated using SD.

## Results

### Activation of microglia by prenatal VPA exposure was involved in neural circuit dysfunction and post-developmental behavioral disorders

Pregnant mice were orally administered VPA on embryonic day 11.5 (E11.5) (the protocol is shown in Fig. 1), and the VPA concentration in the maternal serum increased to approximately 73 μg/mL, which is almost equivalent to the therapeutic range in humans (from 50 to 100 μg/mL), 3 h after treatment (Additional file 1: Fig. S1). VPA was detected in the fetal brain 3 h but not 24 h after administration (Additional file 1: Fig. S1), indicating that VPA could be transiently translocated into the fetal brain and then excreted immediately. Neural development, as evaluated by eye opening, was delayed in VPA-treated mice (Fig. 2A). Although fetal VPA exposure did not affect exploratory behavior or locomotor activity (Fig. 2B), VPA decreased the alternation, which is an index of spatial working memory defects, and decreased the amount of time spent exploring around mice, indicating abnormal social affiliation (Fig. 2C and D). VPA-treated mice also showed an increase in the number of buried marbles (Fig. 2E), suggesting that prenatal VPA exposure elicited repetitive behaviors. The number of microglia in the hippocampal CA1, CA3 and DG regions was similar between postnatal day 10 (P10) vehicle-treated mice and VPA-treated mice (Fig. 2F and G). However, the microglial soma area was significantly increased in the CA1 region by prenatal VPA exposure, and CD68 protein levels were also increased in VPA-treated mice (Fig. 2F, H and I). Flow cytometry showed that peripheral macrophages did not invade the hippocampus in P10 VPA-treated mice (data not shown). Prenatal VPA exposure showed no change in the expression of glial fibrillary acidic protein (GFPA) and S100β in the hippocampus of P10 mice, suggesting that VPA does not affect astrocytic activity (Additional file 1: Fig. S2). Thus, these data clearly showed that prenatal exposure to VPA can activate microglia in the hippocampal CA1 region during the early postnatal periods.

There was no change in microglial number, soma size or CD68 expression in the hippocampal CA1 region of VPA-treated mice at the age of 6 weeks (Additional file 1: Fig. S3A–D). However, when basal neuronal responses upon electrical stimulation were measured, prenatal VPA treatment significantly increased excitatory activity in the hippocampal CA1 region at 6 weeks of age (Additional file 1: Fig. S3E and F). Therefore, these changes in excitation/inhibition balance in the hippocampus might contribute to abnormal behavior elicited by VPA treatment.

Minocycline (7-dimethylamino-6-dimethyl-6-deoxytetracycline), a second-generation semisynthetic tetracycline analog, is widely used to prevent the activation of microglia [41]. When minocycline was administered from P1 to P10 via lactation, the minocycline concentrations in the maternal serum and the hippocampi of the pups at P10 were 1.20 ± 0.10 μg/mL and 0.56 ± 0.05 μg/g tissue, respectively, indicating that minocycline was accumulated in the pup brain when administered via lactation. Iba1/CD68 staining showed that microglial activation, such as soma enlargement and increased CD68 expression in the CA1 region, induced by VPA was largely suppressed by minocycline administration (Fig. 3A–D). Importantly, behavioral disorders induced by VPA, such as defects in social affiliation and repetitive behaviors, were also alleviated by minocycline treatment via lactation from P1 to P21 (Fig. 3F and G). Unfortunately, since treatment with minocycline alone exacerbated special working memory (Fig. 3E), we could not evaluate the effects of activated microglia on special working memory. Minocycline has been reported to have multiple functions in the brain, including the action into the endocannabinoid system and the inhibition of dopamine release [42, 43]. In addition, our unpublished data showed that hippocampal levels of brain-derived neurotrophic factor were largely decreased by minocycline treatment according to our experimental protocol described here (data not shown). Therefore, these actions of minocycline in addition to microglial suppression might be involved in the deterioration of special working memory.

Basal neuronal responses in the three major synaptic connections in the hippocampus (CA3-CA1, Schaffer collateral afferent; DG-CA3, mossy fibers; EC-DG, perforant pathway) were measured upon electrical stimulation, and it was found that prenatal VPA exposure significantly increased excitatory activity, especially upon stimulation of Schaffer collaterals, in P10 mice and that this excitation–inhibition imbalance was clearly suppressed by minocycline treatment (Fig. 4A, B). VPA treatment tended to elicit an excitation–inhibition imbalance upon mossy and perforant stimulation, but the effect was not significant (Fig. 4C–F). Taken together, these results indicate that microglial activation during development elicited by fetal VPA exposure can cause neural circuit dysfunction and behavioral disorders after development. As shown in Additional file 1: Fig. S2E and F, excitation–inhibition imbalance in the CA1 region of VPA-treated mice remained until the age of 6 weeks, when behavioral abnormalities were detected. Therefore, overexcitation in the hippocampus during the developmental stage might cause abnormal post-growth behaviors.

### CCL3 expression was upregulated in microglia during development in an epigenetic-dependent manner

**Fig. 5:**
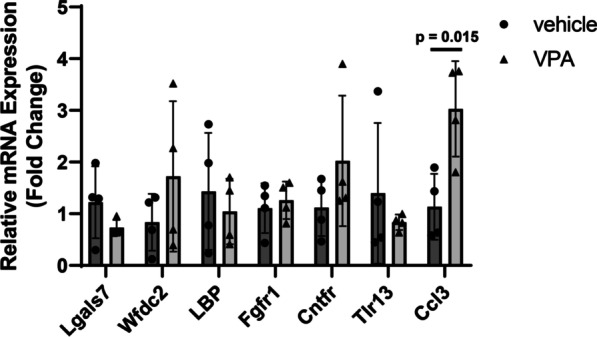
Confirmation of candidate gene expression by qPCR. VPA (800 mg/kg) was orally administered on E11.5. The hippocampus was isolated from male P10 pups, total RNA was extracted, and qPCR was performed. The values are presented as the mean ± S.D. (*n* = 4 male pups from 2 dams in each group). The data were analyzed using Student's *t* test

VPA inhibits histone deacetylase in addition to GABA transaminase [44]. Therefore, gene expression in the hippocampi of P5 vehicle- and VPA-treated mice was analyzed by CAGE-seq. Genes that were upregulated 4 times or downregulated 4 times in the VPA-treated group compared with the vehicle-treated group and with p values lower than 0.1 were extracted, and genes reportedly expressed in the brain were selected (Additional file 1: Table S3). The expression of the 7 selected genes (*lgals7*, *wfdc2*, *lbp*, *fgfr1*, *cntfr*, *tlr13* and *ccl3*) was confirmed by real-time PCR. CCL3 mRNA levels showed significant increases in the hippocampi of VPA-treated mice compared with those of vehicle-treated mice (Fig. 5); thus, we focused on the role of CCL3 in prenatal VPA exposure-induced developmental defects.

**Fig. 6:**
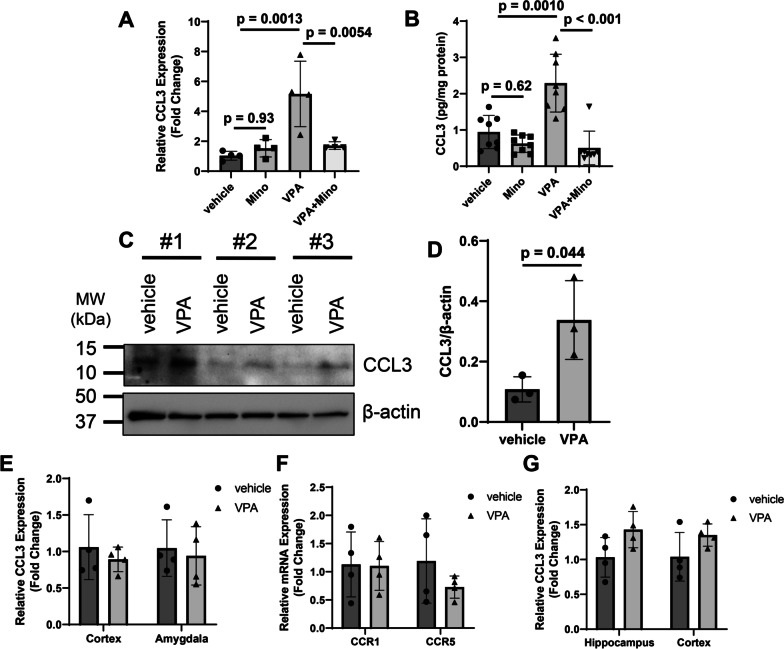
CCL3 expression upregulation in microglia during development induced by prenatal VPA exposure. VPA (800 mg/kg) was orally administered on E11.5. Minocycline (100 mg/kg) was administered to dams by drinking water from P1 to P10. **A** Total RNA was extracted from the hippocampus, and CCL3 mRNA levels were determined by qPCR. The values are presented as the mean ± S.D. (*n* = 4 male pups from 2 dams in each group). The data were analyzed using two-way ANOVA [*F*(1, 12) = 11.82, *p* = 0.0049] followed by Tukey's *t* test. **B** CCL3 protein expression in the hippocampus was measured by ELISA. The values are presented as the mean ± S.D. (*n* = 8 male pups from 2 dams in each group). The data were analyzed using two-way ANOVA [*F*(1, 28) = 15.46, *p* = 0.0005] followed by Tukey's *t* test. **C**, **D** CD11b-positive cells were isolated from the hippocampi of male P10 mice, and then CCL3 levels were measured by western blotting. Representative images are shown in panel **C**, and the results of quantitative analysis of the bands are presented in panel **D**. Hippocampi collected from 5 male pups were used to isolate CD11b-positive cells. Three independent experiments were performed with 5 litters (15 male vehicle pups from 3 dams and 15 male VPA pups from 3 dams were used for this experiment). The values are presented as the mean ± S.D. The data were analyzed using Student's *t* test. **E** CCL3 mRNA expression in the cortex and amygdala in P10 mice was evaluated by qPCR. **F** CCR1 and CCR5 mRNA levels in the hippocampi of P10 mice were measured. **G** CCL3 mRNA expression was measured in the P1 mouse brain. The values are presented as the mean ± S.D. The data were analyzed using Student's *t* test

The increase in CCL3 mRNA expression in the hippocampus of P10 mice induced by prenatal VPA treatment was significantly suppressed by minocycline administration, and the increase in CCL3 protein expression induced by VPA was also attenuated by minocycline (Fig. 6A and B). Next, CD11b-positive cells, mainly microglia in the brain, were immunoprecipitated from the P10 hippocampi, and CCL3 protein expression was evaluated by western blotting. CCL3 expression in CD11b-positive cells was significantly increased by prenatal VPA exposure (Fig. 6C and D), suggesting that CCL3 expression is upregulated in hippocampal microglia at least after birth. CCL3 mRNA levels in the cortex and amygdala were not affected by VPA (Fig. 6E). No change in the expression of CCR1 and CCR5, which are receptors for CCL3, was observed between vehicle- and VPA-treated mice (Fig. 6F). In addition, CCL3 expression was not increased in the hippocampi of P1 mice (Fig. 6G).

The maternal inflammatory state can influence neonates through a process known as maternal immune activation (MIA) [45]; however, VPA administration did not cause an increase in the serum levels of proinflammatory cytokines such as IL-1β and IL-6 (Additional file 1: Fig. S4). Therefore, MIA is unlikely to contribute to developmental microglial activation in this VPA exposure model. CCL3 was reported to be upregulated epigenetically in macrophages, and the upstream region of the CCL3 gene has a fundamental role in the upregulation [46]. Thus, to identify the mechanism by which CCL3 expression is upregulated in the hippocampus by VPA treatment, chromatin immunoprecipitation (ChIP) was performed. DNA was extracted from CD11b-positive cells in the hippocampus and precipitated with anti-histone H3 and H4 antibodies and anti-acetyl-histone H3 and H4 antibodies. Real-time PCR was performed using a primer designed upstream of the CCL3 gene, according to the report by Kiguchi et al. [46]. More of the upstream region of the CCL3 gene was detected in precipitated DNA with anti-acetyl-histone H3 prepared from VPA-treated mice than vehicle-treated mice (Additional file 1: Fig. S5A), suggesting that histone H3 upstream of the CCL3 gene can be acetylated in the hippocampal microglia of VPA-treated mice. Western blotting confirmed that histone H3 acetylation in the hippocampus increased in the VPA-treated group compared with the vehicle-treated group (Additional file 1: Fig. S5B and C). Taken together, these results suggest that CCL3 expression may be upregulated via an epigenetic mechanism that includes histone H3 hyperacetylation, at least in part.

### Antagonism of CCR5 after birth suppressed neural circuit dysfunction and behavioral disorders induced by prenatal VPA exposure

**Fig. 7:**
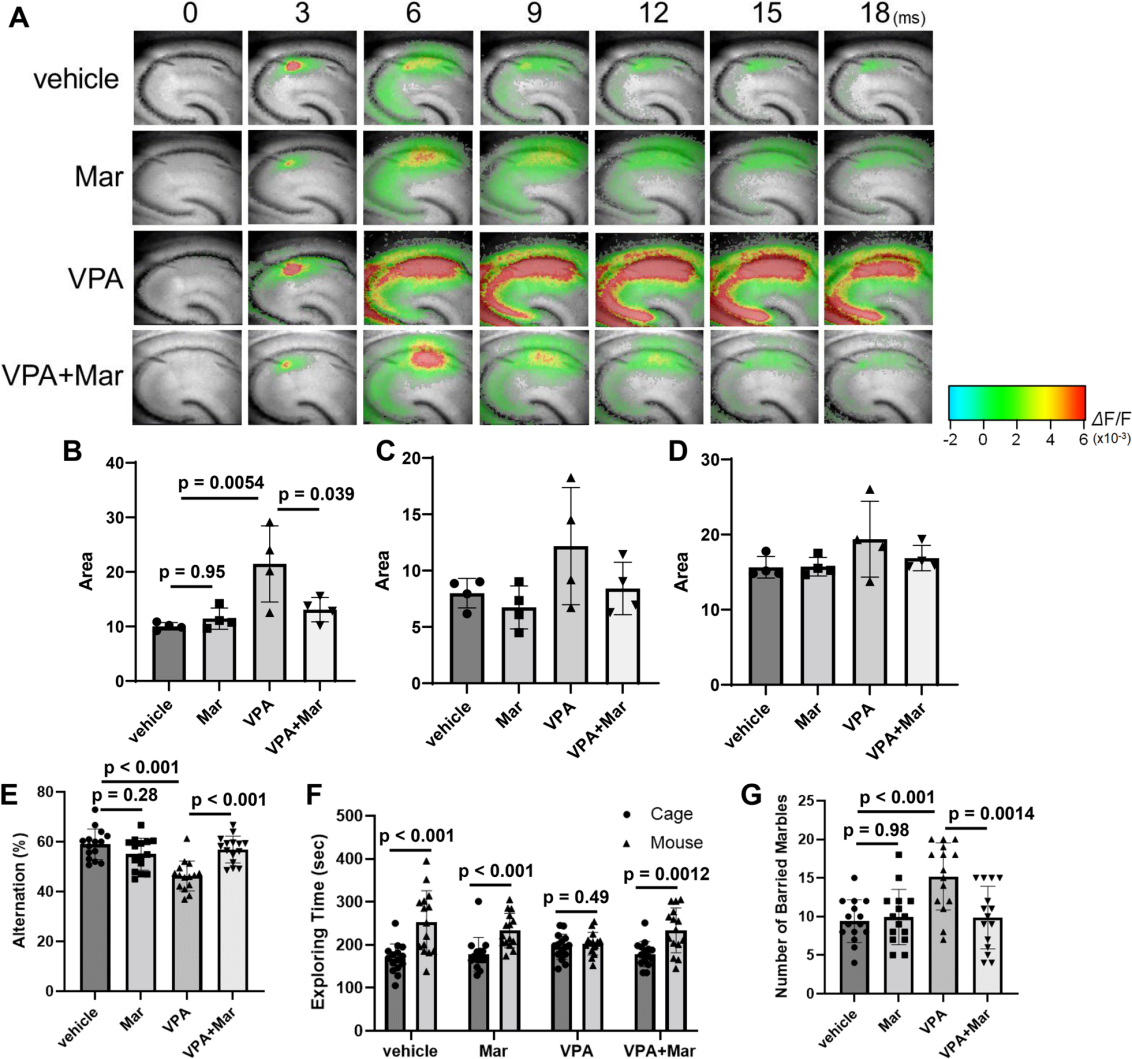
Suppression of VPA-induced excessive neuronal activity and behavioral disorders by postnatal maraviroc treatment. VPA (800 mg/kg) was orally administered on E11.5. Maraviroc (80 mg/kg) was administered to dams by drinking water from P1 to P21. **A**–**D** Electrical stimulation was applied to Schaffer collateral afferents, the mossy fiber pathway and the molecular layer in hippocampal slices from P10 mice. **A** Representative images showing activity in the CA1 region. **B**–**D** Quantification of the neural response after stimulation of Schaffer collateral afferents (**B**), the mossy fiber pathway (**C**) and the molecular layer (**D**). The values are presented as the mean ± S.D. (*n* = 4 male pups from 2 dams in each group). The data were analyzed using two-way ANOVA [**B** *F*(1, 12) = 6.602, *p* = 0.0246, **C** *F*(1, 12) = 0.6564, *p* = 0.4336, **D** *F*(1, 12) = 0.8452, *p* = 0.3760] with Tukey's corrected multiple comparison tests. **E** The number of alternations in the Y-maze test. The values are presented as the mean ± S.D. (*n* = 15 male pups from 3 dams in each group). The data were analyzed using two-way ANOVA [*F*(1, 56) = 22.8, *p* < 0.0001] with Tukey's corrected multiple comparison tests. **F** Social interaction, as measured by exploration time in the open field. The values are presented as the mean ± S.D. (*n* = 15 male pups from 3 dams in each group). The data were analyzed using Student's *t* test. **G** The number of buried marbles. The values are presented as the mean ± S.D. (*n* = 15 male pups from 3 dams in each group). The data were analyzed using two-way ANOVA [*F*(1, 56) = 9.235, *p* = 0.0036] with Tukey's corrected multiple comparison tests

CCR1 and CCR5 are the main receptors for CCL3. Among these, CCR5 is known to be largely distributed in the brains of several species, including humans and mice [47]. Thus, we attempted to alleviate neural circuit dysfunction and abnormal behaviors induced by fetal VPA exposure with a CCR5 antagonist, maraviroc (UK-427857). When maraviroc was administered via lactation from P1 to P10, the maraviroc concentrations in the maternal serum and hippocampi of the pups were 92.7 ± 10.0 ng/mL and 331 ± 53 ng/g tissue, respectively, indicating that maraviroc was able to enter the brains of the pups. Maraviroc did not affect microglial activity in the hippocampus of mice prenatally exposed to VPA (data not shown). Maraviroc clearly suppressed the increase in excitability induced by prenatal VPA exposure in the hippocampal CA1 region of P10 mice (Fig. 7A–D). Maraviroc administration also suppressed VPA-induced post-developmental defects in spatial working memory and social affiliation and repetitive behaviors (Fig. 7E–G). Therefore, maraviroc can recover post-developmental behavioral disorders by alleviating the excitation–inhibition imbalance during development elicited by prenatal VPA treatment.

**Fig. 8:**
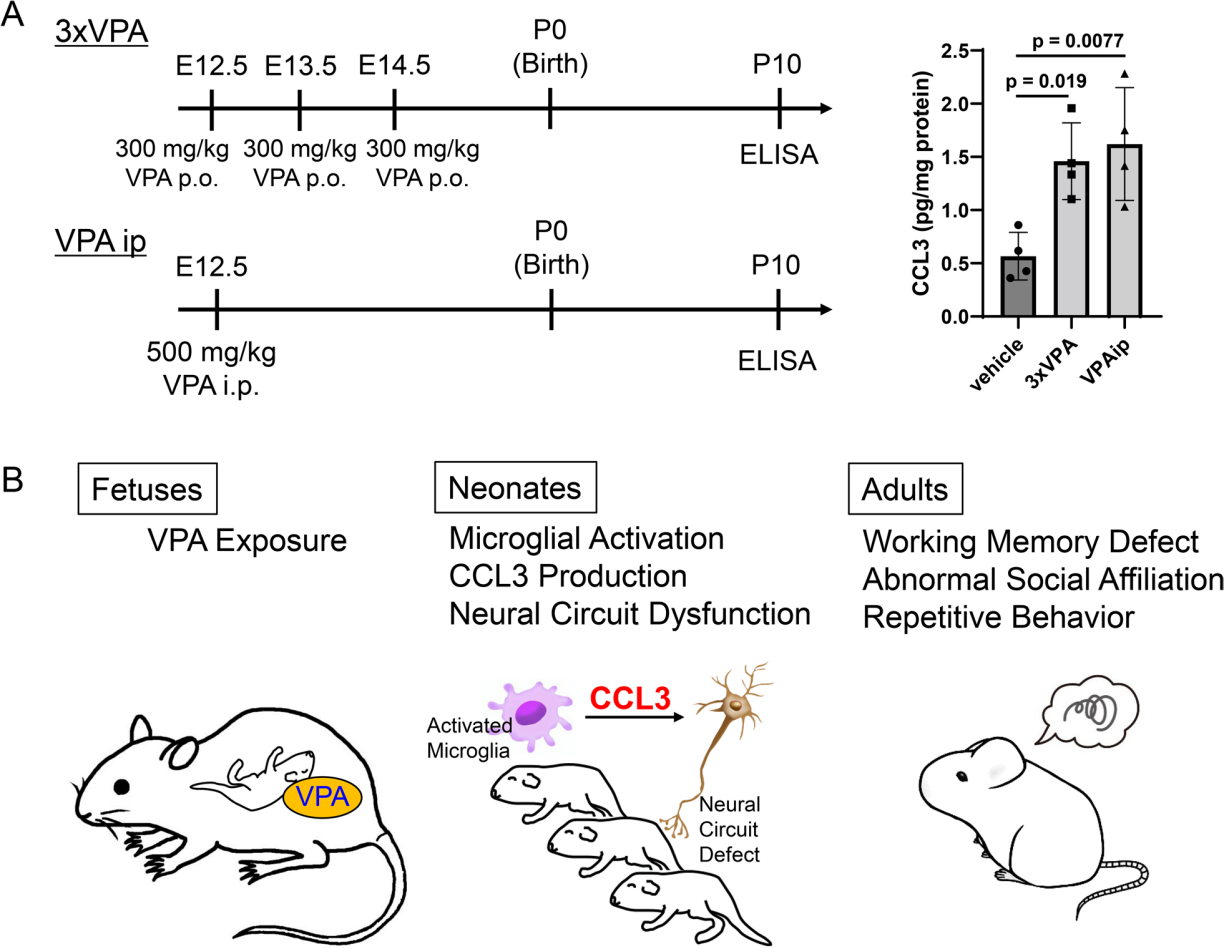
The putative mechanism by which prenatal VPA exposure elicits post-developmental behavioral disorders. **A** CCL3 expression upregulation in the hippocampus during development elicited by several VPA administration protocols. VPA (300 mg/kg) was orally administered three times, i.e., at E12.5, E13.5 and E14.5 (3xVPA), or VPA (500 mg/kg) was intraperitoneally administered at E12.5 (VPAip). CCL3 protein expression in the hippocampi of P10 mice was determined by ELISA. The values are presented as the mean ± S.D. (*n* = 4 male pups from 4 dams in each group). The data were analyzed using one-way ANOVA [*F*(2, 9) = 8.387, *p* = 0.0088] with Dunnett's corrected multiple comparison test. **B** Schematic showing the action of CCL3 in mice with fetal VPA exposure

Several VPA administration protocols are used to study prenatal VPA-induced developmental disorders [8, 18]. Thus, CCL3 expression in the hippocampus was assessed after VPA administration rather than at the dose of 800 mg/kg VPA orally on E11.5. Three applications of 300 mg/kg VPA orally at E12.5, 13.5 and 14.5 to dams significantly increased hippocampal CCL3 levels at P10 (Fig. 8A). Intraperitoneal treatment with 500 mg/kg VPA at E12.5 in dams also increased CCL3 expression (Fig. 8A). Therefore, CCL3 upregulation in the developing hippocampus is considered one of the general mechanisms by which prenatal exposure to VPA induces post-developmental behavioral disorders.

## Discussion

Several reports have shown that microglia are activated by fetal VPA exposure, although the characteristics of microglial activation are different [14–16]. Our results showed that microglial morphology was altered, soma enlargement was observed and microglial CD68 expression increased in the hippocampal CA1 region upon prenatal exposure to VPA, clearly indicating microglial activation after birth induced by prenatal VPA treatment. VPA was detected in the fetal brain 3 h after administration but not 24 h after treatment. Therefore, certain changes in the fetal brain induced by VPA affect microglia in the developmental stage. VPA can act on GABA transmission but is also known to be a potent HDAC inhibitor [44]. Generally, HDAC inhibitors exert neuroprotective effects [48]. In microglia, HDAC inhibitors have been reported to affect gene expression to alter the microglial phenotype toward protective M2 via the GSK3β/PTEN/Akt pathway [49]. In this study, hyperacetylation of histone H3 was detected in microglia of VPA-treated mice in the early postnatal periods. Therefore, epigenetic changes might be induced due to the inhibition of HDAC activity in VPA-treated mice. Because microglia are derived from yolk sac macrophages and enter the brain on E9.5–E10.5 [50], it is possible that fetal microglia are directly affected by VPA. However, considering that CCL3 expression showed no change at P1 but increased at P10, changes in the fetal period are not persistent just after birth. Namely, alteration of gene expression can occur in the early postnatal period from P1 to P5 when CCL3 upregulation was detected by CAGE-seq. Further study is needed to uncover how fetal VPA exposure changes the gene expression of microglia after birth.

When microglial activation was suppressed by minocycline, neural circuit and behavioral dysfunctions were largely alleviated. Thus, microglial activation during development can cause the formation of abnormal neural circuits and several post-developmental behavioral disorders. This is supported by evidence that microglial activation is observed in patients with autism [51] and attention-deficit/hyperactivity disorder [52]. Microglia actively engulf synapses as well as spines and thus play a major role in synaptic pruning during postnatal development to contribute to the maturation of neural circuits [53]. In addition, pruning of excess synapses by microglia mediates synapse loss in Alzheimer's disease [11]. Therefore, engulfment of synapses by microglia may be involved in development and pathology; excess activation of microglia could decrease the number of synapses and spines. However, in a VPA model, an increased spine density of excitatory neurons was observed [54]. Another VPA model showed that an increase in the number of excitatory synapses was detected at P3, which persisted until P35 [55]. Therefore, the relationship between microglial activity and the number of synapses/spines is controversial, at least in prenatal VPA-treated models. Based on this background, we focused on a gene affected by prenatal exposure to VPA.

CCL3, also known as macrophage inflammatory protein-1α (MIP-1α), is a chemokine that is involved in the migration of immune cells via its specific receptors CCR1 and CCR5. We found that fetal exposure to VPA increased CCL3 expression in microglia in the developing hippocampus. CCL3 expression is upregulated in macrophages by an epigenetic mechanism after peripheral nerve injury [46]. In this study, ChIP qPCR and immunoblotting revealed that VPA exposure enhanced histone H3 acetylation and that acetylated histone H3 interacted with the CCL3 promoter/enhancer region upstream of the CCL3 transcription start site. Thus, CCL3 expression may be upregulated in microglia during development via an epigenetic mechanism. Kataoka et al. reported that histone hyperacetylation occurs after VPA administration [18], suggesting that VPA exposure upregulates CCL3 expression via an epigenetic mechanism. Importantly, administration of an antagonist of CCR5, maraviroc, clearly alleviated both neural circuit dysfunction and behavioral disorders induced by fetal exposure to VPA. Taken together, these results suggest that CCL3 derived from activated microglia during development could be involved in the development of abnormal post-developmental behaviors induced by prenatal exposure to VPA.

Marciniak et al. reported that CCL3 reduced basal synaptic transmission at Schaffer collateral–CA1 synapses without affecting NMDA receptor-mediated field potentials and concluded that CCL3 is a hippocampal neuromodulator that is able to regulate synaptic plasticity mechanisms [56]. However, our preliminary data showed that CCL3 did not affect long-term potentiation (LTP) in acute hippocampal slices from P10 mice (data not shown). The difference between the previous study and our study is unclear, but CCL3 is thought to affect processes other than synaptic transmission. We found the overexcitation in the hippocampus during the developmental stage induced by prenatal VPA administration. It is reported that fetal VPA exposure reduced the number of parvalbumin-positive inhibitory neurons, leading to neural circuit defect [57]. Microglia stimulated with CCR5 showed high migration activity [58], and microglia moved into the synaptic cleft of inhibitory synapses to suppress inhibitory synaptic transmission [59]. In addition, microglial CCR5 could induce M2 phagocytic microglia differentiation, which may elicit neuronal pyroptosis and neurological deficits [60, 61]. These may explain excitation/inhibition imbalance in the hippocampus CA1 region of VPA-exposed mice.

CCR5 expressed in neurons is reported to be involved in learning and memory [62]. CCR5 activation via its ligand CCL3 impairs performance of social recognition and passive avoidance [63, 64]. CCR5 is a seven transmembrane, G protein-coupled receptor and can activate multiple kinase signaling such as phosphoinositide-3 kinase, mitogen-activated protein kinases and protein kinase C. Among the downstream signals of CCR5, cAMP responsive element binding protein (CREB) is known to be required for a variety of complex forms of memory [65]. Compared to wild-type mice after learning, there was a significant enhancement in CREB phosphorylation in the dorsal hippocampus in *Ccr5* knockout mice after learning [64], suggesting that CCR5 is involved in CREB phosphorylation and that CCR5 impacts learning and memory via CREB signaling. In addition, stimulation of CCR5 reportedly increased Ca^2+^ concentration in neurons [66], suggesting that Ca^2+^ signaling is also involved in learning and memory. On the other hand, a role of CCR5 in astrocytes is largely unclear, although astrocytic CCR5 is reported to activate astrocytes to promote inflammatory reaction [67, 68]. In this study, however, astrocytic activation was not detected, suggesting that microglial CCL3 does not modulate astrocytic activity.

CCR1 and CCR5 are expressed at the highest levels in oligodendrocyte precursor cells (OPCs) in the embryonic spinal cord and postnatal brain, respectively [69]. Stimulation of purified rat OPCs with CCL3 leads to a decrease in the migration of OPCs without affecting proliferation [70]. Notably, the number of NG2- and O4-positive oligodendrocytes is reduced in CCR5 knockout mice with experimental autoimmune encephalomyelitis, most likely due to decreased demyelination [71]. Considering these findings, CCL3 released from microglia during development might act on OPCs and induce subsequent myelination to suppress the formation of neural circuits. The direct role of CCL3 in OPCs during development remains to be addressed.

CCL3 expression in the hippocampus has been reported to be upregulated in rodents upon exposure to propofol and toluene during development [72, 73]. Notably, the plasma CCL3 concentration in autistic children was significantly higher than that in age-matched typically developing children [74]. Maraviroc treatment has been reported to suppress hyperlocomotion induced by cocaine administration [75]. Based on these findings, modification of neural circuits by CCL3 may be one of general mechanisms underlying behavioral disorders. In addition, as shown in this study, early intervention with maraviroc during the lactation period improved developmental disorders. Recently, a microRNA, miR-21, was reported to decrease CCL3 expression [76]. Thus, microRNAs might be candidates for suppressing CCL3 signaling, which is involved in neural circuit dysfunction. Several tools, such as antagonists and microRNAs, may be useful for the treatment of developmental disorders.

## Conclusion

Prenatal VPA exposure-induced increases in CCL3 expression in microglia during development can cause neural circuit dysfunction and post-developmental behavioral disorders (Fig. 8B). Administration of the CCR5 agonist maraviroc during lactation clearly suppresses behavioral disorders induced by VPA. CCR5 antagonists might be effective in treating developmental disorders in which CCL3 is involved.

## Supplementary Information

**Additional file 1. Fig. S1** to **S5**, **Tables S1** to **S4** and related methods.

**Additional file 2. Figs. S6** to **S9** that show whole membrane images of immunoblotting.

## Data Availability

The CAGE-seq data have been deposited in the Gene Expression Omnibus (GEO) database (accession no. GSE180564). All other data supporting the conclusions of this article are included within the article and its supplemental file.

## Abbreviations

CAGE-seq: Cap analysis gene expression sequencing
CCL3: C–C motif chemokine ligand 3
ChIP: Chromatin immunoprecipitation
DG: Dentate gyrus
GABA: Gamma-aminobutyric acid
GFAP: Glial fibrillary acidic protein
IHC: Immunohistochemistry
MIA: Maternal immune activation
OPC: Oligodendrocyte precursor cell
VPA: Valproic acid
VSD: Voltage-sensitive dye
