# Supplementary material for: A CCR5 antagonist, maraviroc, alleviates neural circuit dysfunction and behavioral disorders induced by prenatal valproate exposure
Source: J Neuroinflammation. 2022 Jul 29;19:195. doi: 10.1186/s12974-022-02559-y (PMC9335995; doi:10.1186/s12974-022-02559-y)
Supplement: Supplementary file 1 — Additional file 1. Fig. S1 to S5, Tables S1 to S4 and related methods. [file 12974_2022_2559_MOESM1_ESM.docx]

**Additional file 1**

*Ishihara et al.* A CCR5 antagonist, maraviroc, alleviates neural circuit dysfunction and behavioral disorders induced by prenatal valproate exposure.

**Supplemental Methods**

**VPA level determination by HPLC-UV**

Minocycline level determination was performed according to a previous report (Anal Sci 1(4):385-387, 1985). Serum samples, brain homogenates or VPA standards were mixed with an equal volume of acetonitrile using a vortex mixer. After centrifugation, 20 µL of the supernatant was injected into an ODS-3 column (3.0 × 250 mm, 4 µm) (GL Science, Tokyo, Japan), and 400:600:1 acetonitrile:water:trifluoroacetic acid was used as the mobile phase. The flow rate was 0.5 mL/min, the column temperature was 30°C, and UV detection was performed at 220 nm.

**Chromatin immunoprecipitation (ChIP)**

Microglia isolated from the hippocampus using CD11b magnetic beads (Cells 10(4):718, 2021) were fixed in 1% formaldehyde for 10 min at room temperature, and immunoprecipitation was performed with the antibodies listed in Table S1 using the Pierce Agarose ChIP Kit (Thermo Fisher Scientific) according to the manufacturer’s instructions (Toxicol Sci 170(2):404-414, 2019). The resulting immunoprecipitates, including DNA, were analyzed by qPCR. The primers that were used are listed in Table S4. The percentage input of each sample was calculated from the Ct values.

**Determination of serum cytokine concentrations**

Blood was collected from the tail vein of dams or from the inferior vena cava of male P10 pups. The serum was isolated, and then cytokine concentrations in the serum were determined with a LEGENDplex Mouse Inflammation Panel (BioLegend) using a CytoFREX S flow cytometer (Beckman Coulter K.K., Tokyo, Japan) according to the manufacturers’ instructions.

**Table S1. Antibodies used in this study**

Target Source RRID Dilution (utility)

Iba1 Wako Pure Chemical AB_839504 1/400 (IHC)

CD68 BIO-RAD AB_322219 1/400 (IHC)

CCL3 SCB AB_10614671 1/500 (WB)

β-actin SCB AB_2714189 1/5,000 (WB)

GFAP Agilent (Dako) AB_10013482 1/400 (IHC)

Histone H3 CST AB_1642229 1/2,000 (WB, ChIP)

Ac-Histone H3 (Lys9) CST AB_823528 1/1,000 (WB, ChIP)

Histone H4 CST AB_1147658 - (ChIP)

Ac-Histone H4 (Lys8) CST AB_2248400 - (ChIP)

Rat IgG, Alexa 488 TFS AB_2534074 1/200 (IHC)

Rabbit IgG, Alexa 488 TFS AB_10563748 1/200 (IHC)

Rabbit IgG, Alexa 568 TFS AB_10563566 1/200 (IHC)

Mouse IgG, HRP TFS AB_228307 1/10,000 (WB)

Rabbit IgG, HRP TFS AB_228341 1/10,000 (WB)

SCB: Santa Cruz Biotechnology, CST: Cell Signaling Technology, TFS: Thermo Fisher Scientific, ICH: immunohistochemistry, WB: western blotting, FCM: flow cytometry.

**Table S2. Primers used for qPCR**

Name Sequence (5’-3’)

Mouse β-actin-For CTAGGCACCAGGGTGTGATG

Mouse β-actin-Rev GGGGTACTTCAGGGTCAGGA

Mouse Ccl3-For CCATATGGAGCTGACACCCC

Mouse Ccl3-Rev TCAGGAAAATGACACCTGGCT

Mouse CCR1-For AGGTTGGGACCTTGAACCTTG

Mouse CCR1-Rev ACAGTGAGTCTGTGTTTCCAGA

Mouse CCR5-For AAGAGACTCTGGCTCTTGCAG

Mouse CCR5-Rev GAGCTGAGCCGCAATTTGTT

Mouse Cntfr-For GGATCCGGCCGCAGTG

Mouse Cntfr-Rev CCAGACCAAAGTCTTCCTGGG

Mouse Fgfr1-For GTGTGGAAGGTGCACTGGAT

Mouse Fgfr1-Rev CCAGGCTTCTGACCGATTGT

Mouse GFAP-For AGGCGAAGAAAACCGCAT

Mouse GFAP-Rev GGTGAGCCTGTATTGGGACA

Mouse LBP-For GGCTTGGCGTGGTCACTAAT

Mouse LBP-Rev CATCCCGGTAACCTTGCTGT

Mouse Lgals7-For AGCTGAACCACTACCTTGCC

Mouse Lgals7-Rev GTGCTGGGTAGCAGACATGG

Mouse S100β-For TTCCTGGAGGAAATCAAGGAGC

Mouse S100β-Rev GGAAGTCACACTCCCCATCC

Mouse Tlr13-For TGTTTGTCACCTGCTCGGAA

Mouse Tlr13-Rev AATGCCCTCTGCATTGGTGA

Mouse Wfdc2-For TGCTCCAAGCCTAATGGACC

Mouse Wfdc2-Rev GTGGCTTCGTGGAGACTTGA

**Table S3. Candidate genes regulated by prenatal VPA administration**

| Gene name | Log_2_ fold change | p value |
| --- | --- | --- |
| *Lgals7* | -3.1 | 0.027 |
| *Wfdc2* | -2.2 | 0.042 |
| *Lbp* | -3.2 | 0.044 |
| *Fgfr1* | -9.2 | 0.055 |
| *Cntfr* | 11.9 | 0.078 |
| *Tlr13* | -3.9 | 0.078 |
| *Ccl3* | 5.5 | 0.093 |

**Table S4. Primers used for ChIP**

Name Sequence (5’ – 3’)

mCCL3 ChIP Se ACCCAGCAAAAGTTCCAGTC

mCCL3 ChIP An CCCAATCTTTGCACTCCAAG


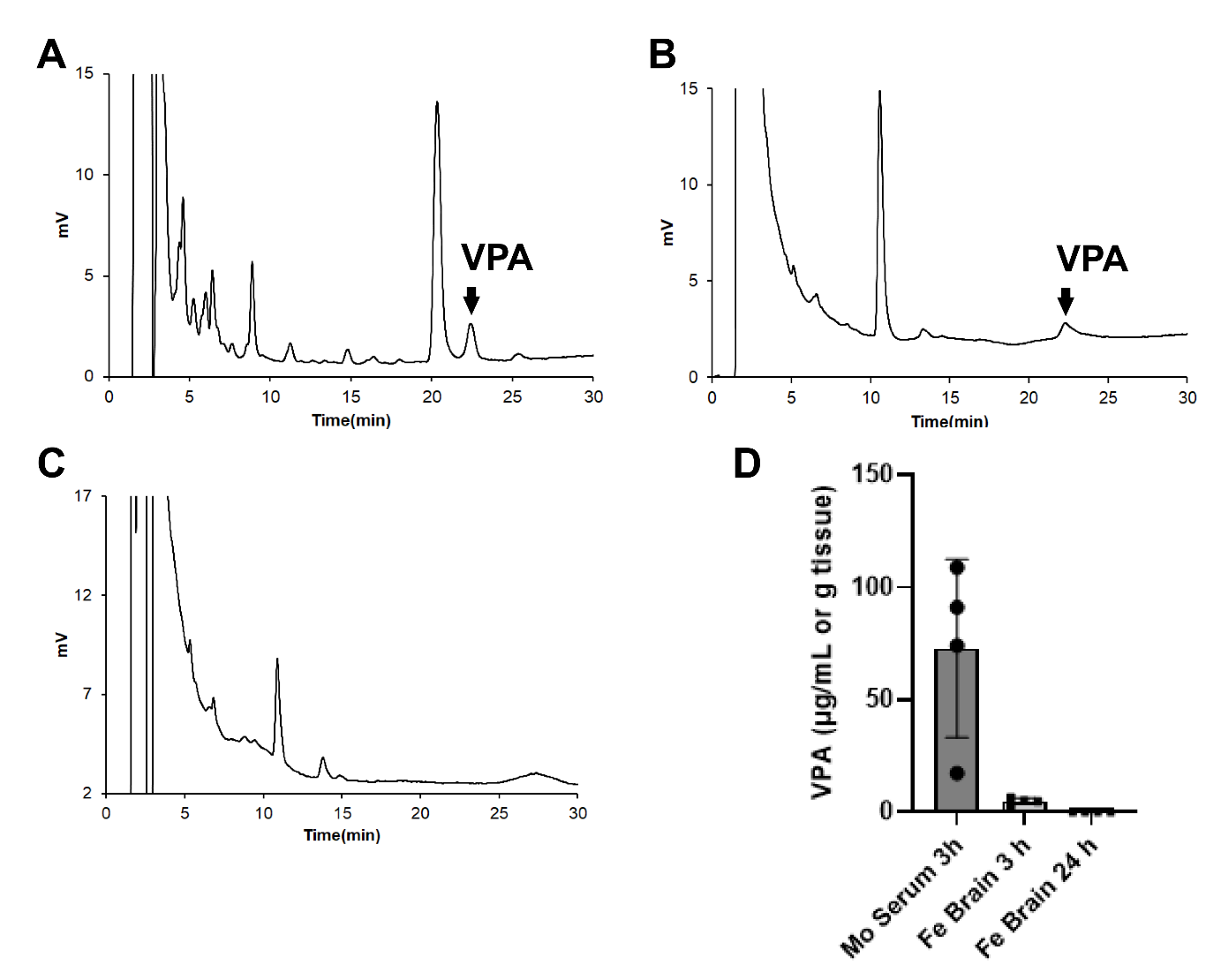


**Figure S1. VPA concentrations in the maternal serum and fetal brain**

Pregnant female mice received a single oral dose of 800 mg/kg VPA on E11.5. VPA concentrations were determined by HPLC-UV. Representative HPLC-UV chromatograms of dam serum 3 h after administration (A) and the fetal brain 3 h (B) and 24 h (C) after treatment. VPA concentrations in the serum and brain tissues are shown in panel D. The values are presented as the mean ± S.D. (n = 4 dams in each group).

**
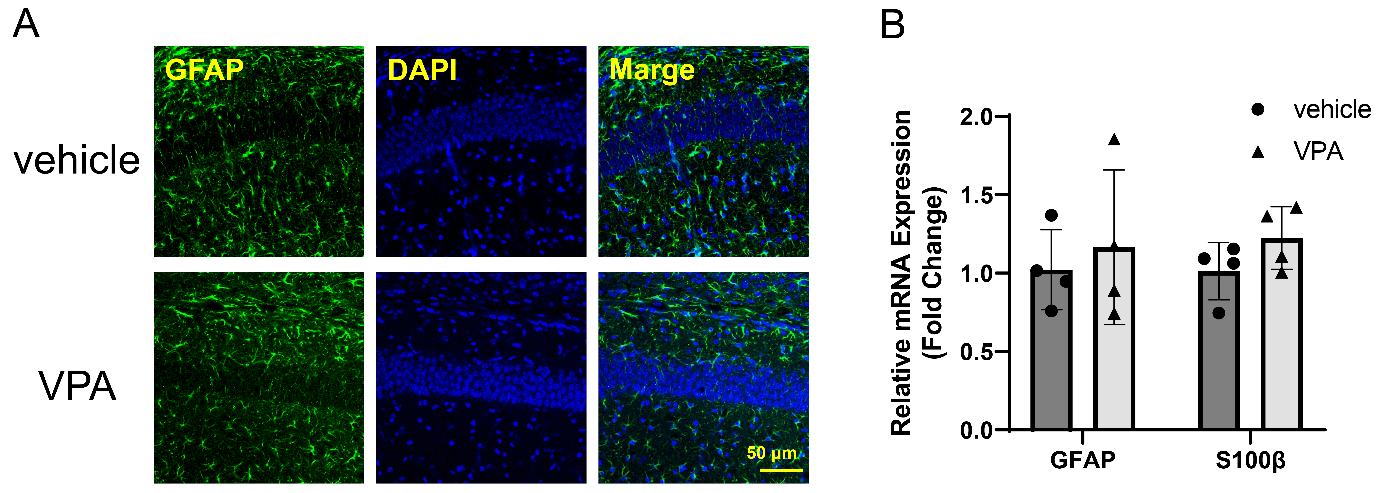
**

**Figure S2. Astrocytic activity in the hippocampi of mice at the age of P10**

Pregnant female mice received a single oral dose of 800 mg/kg sodium valproate on E11.5. (A) Hippocampal slices were prepared from P10 mice and stained for GFAP and DAPI. Representative stained pictures were shown. (B) Total RNA was extracted from the hippocampus, and GFAP and S100β mRNA levels were determined by qPCR. The values are presented as the mean ± S.D. (n = 4 male pups from 2 dams in each group). The data were analyzed using Student's t test.


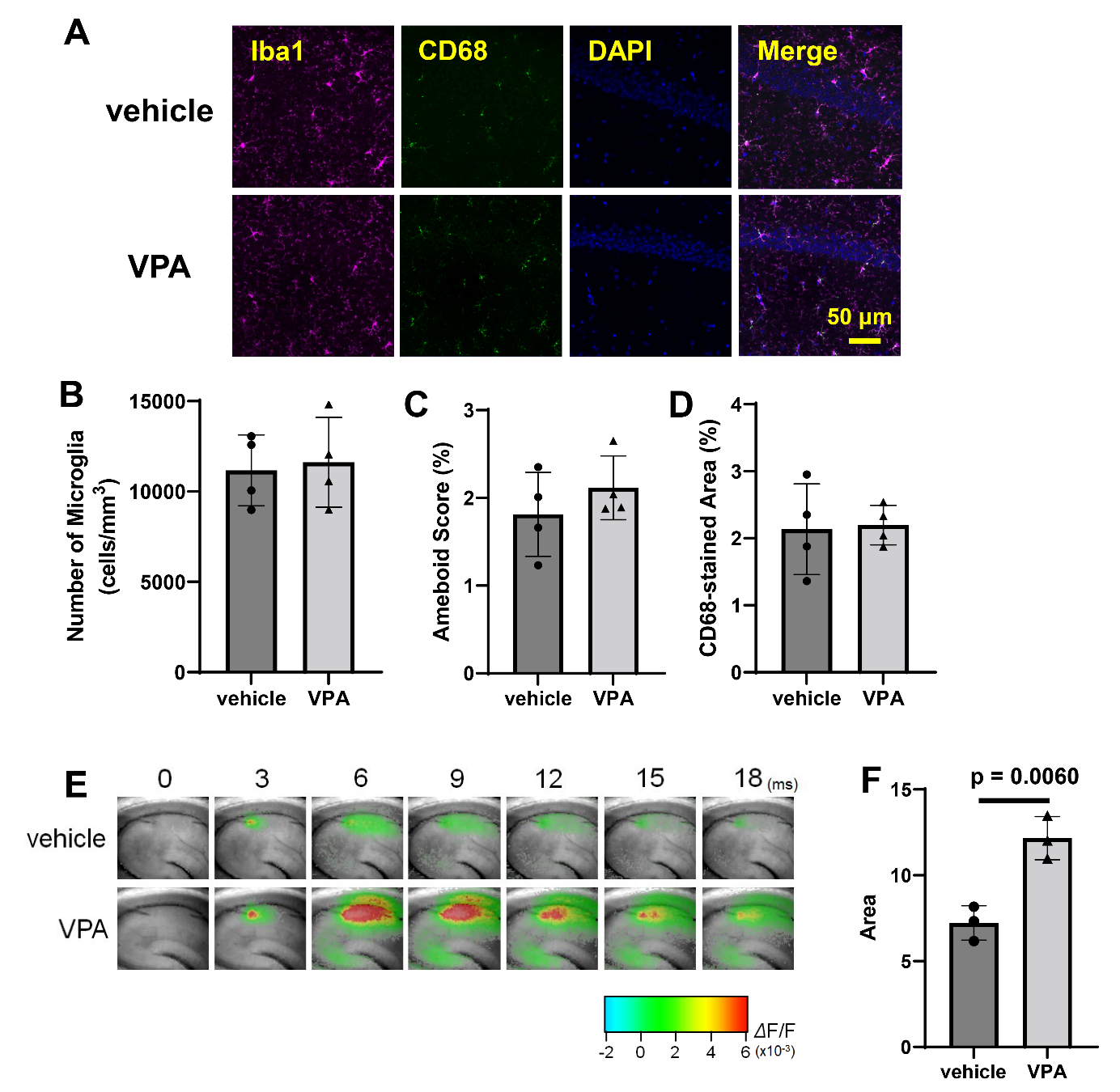


**Figure S3. Microglial activity and neuronal excitation-inhibition imbalance in the hippocampi of mice at the age of 6 weeks**

Pregnant female mice received a single oral dose of 800 mg/kg sodium valproate on E11.5. (A-D) Hippocampal slices were prepared from 6-week-old male pups and stained with Iba1, CD68 and DAPI. (A) Representative images of the CA1 region are shown. (B-D) The number of microglia, the amoeboid score and the CD68-stained area were calculated from the stained images. The values are presented as the mean ± S.D. (n = 4 male pups from 2 dams in each group). The data were analyzed using Student's t test. (E, F) Hippocampal slices were prepared from 6-week-old male pups, and electrical stimulation was applied to Schaffer collateral afferents in the slices. (E) Representative pseudocolored images showing activity. (F) Quantification of the neural response after stimulation. The values are presented as the mean ± S.D. (n = 3 male pups from 2 dams in each group). The data were analyzed using Student's t test.

**
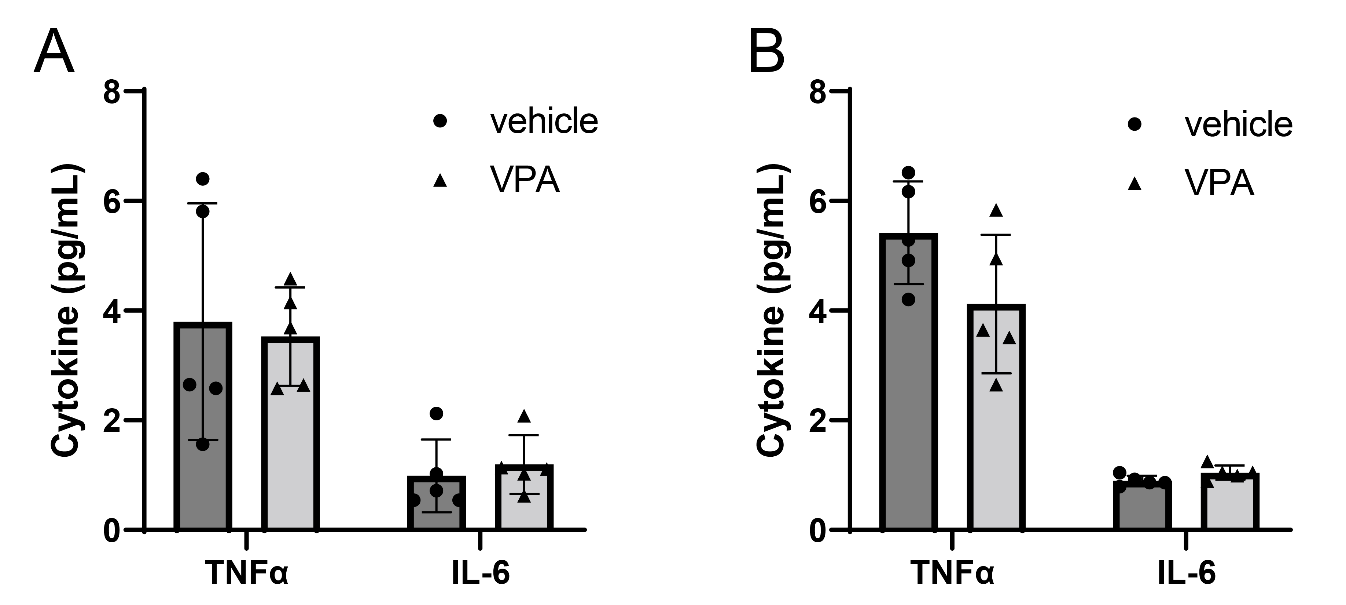
**

**Figure S4. Evaluation of systemic inflammation by measurement of serum inflammatory cytokine levels**

VPA (800 mg/kg) was orally administered on E11.5. Serum was collected from dams 1 day after VPA treatment (A) and male P10 pups (B). The concentrations of TNFα and IL-6 were determined with a LEGENDplex Mouse Inflammation Panel using a flow cytometer. The values are presented as the mean ± S.E. (n = 5 animals, dams or male pups, in each group). The data were analyzed using Student's t test.

**
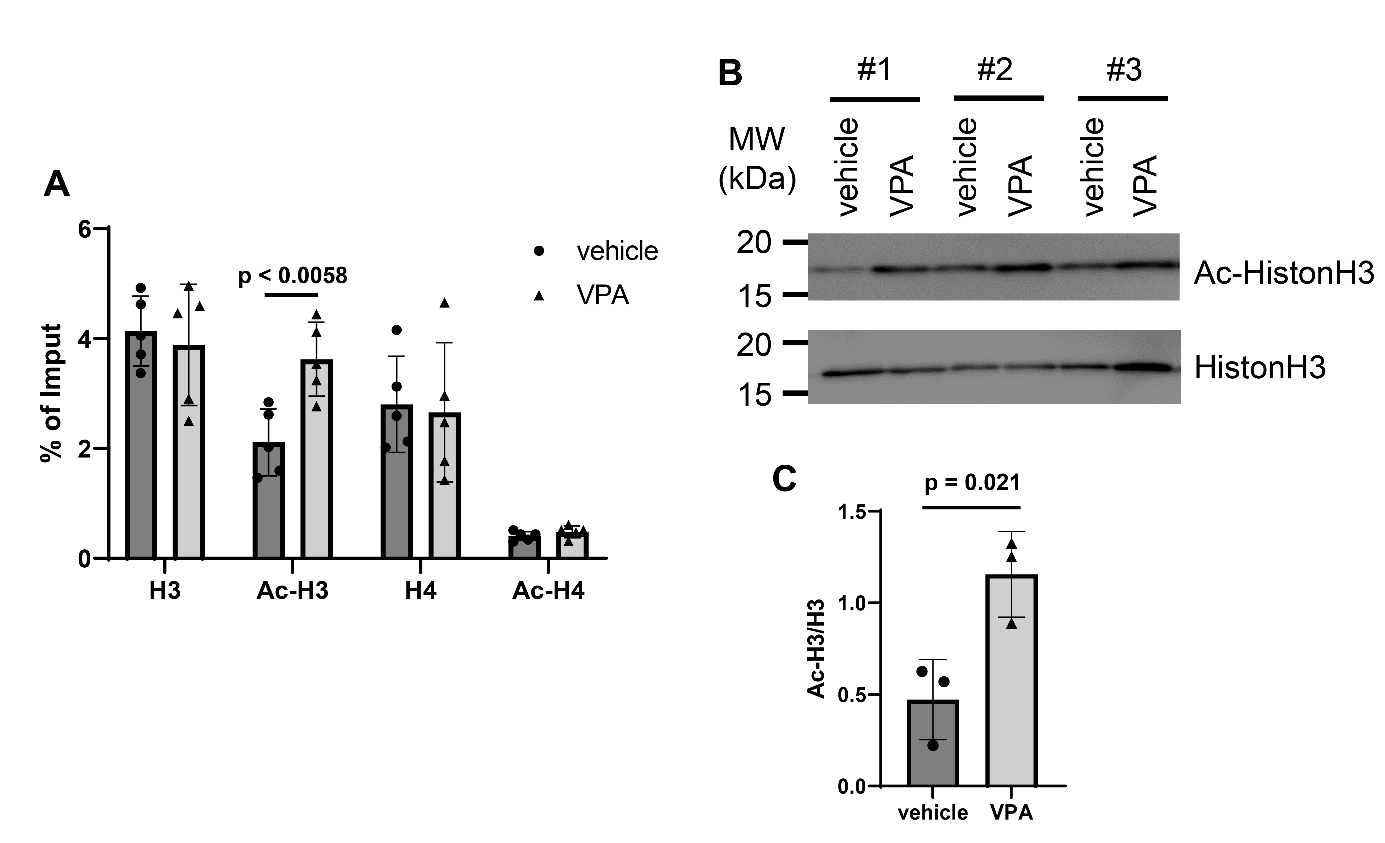
**

**Figure S5. Epigenetic regulation of CCL3 expression in the hippocampi of mice prenatally exposed to VPA**

VPA (800 mg/kg) was orally administered on E11.5. (A) CD11b-positive cells were isolated from the hippocampi of P10 mice, and ChIP was then performed. The values are presented as the mean ± S.D. (n = 5 male pups from 2 dams in each group). The data were analyzed using Student's t test. (B, C) Histone H3 and acetylated histone H3 levels in the hippocampus were measured by western blotting. Representative images are shown in panel B, and the quantification of band intensity is shown in panel C. The values are presented as the mean ± S.D. (n = 3 male pups from 2 dams in each group). The data were analyzed using Student's t test.
