# Supplementary material for: A CCR5 antagonist, maraviroc, alleviates neural circuit dysfunction and behavioral disorders induced by prenatal valproate exposure
Source: J Neuroinflammation. 2022 Jul 29;19:195. doi: 10.1186/s12974-022-02559-y (PMC9335995; doi:10.1186/s12974-022-02559-y)
Supplement: Supplementary file 2 — Additional file 2. Figs. S6 to S9 that show whole membrane images of immunoblotting. [file 12974_2022_2559_MOESM2_ESM.docx]

**Additional file 2**

*Ishihara et al.* A CCR5 antagonist, maraviroc, alleviates neural circuit dysfunction and behavioral disorders induced by prenatal valproate exposure.


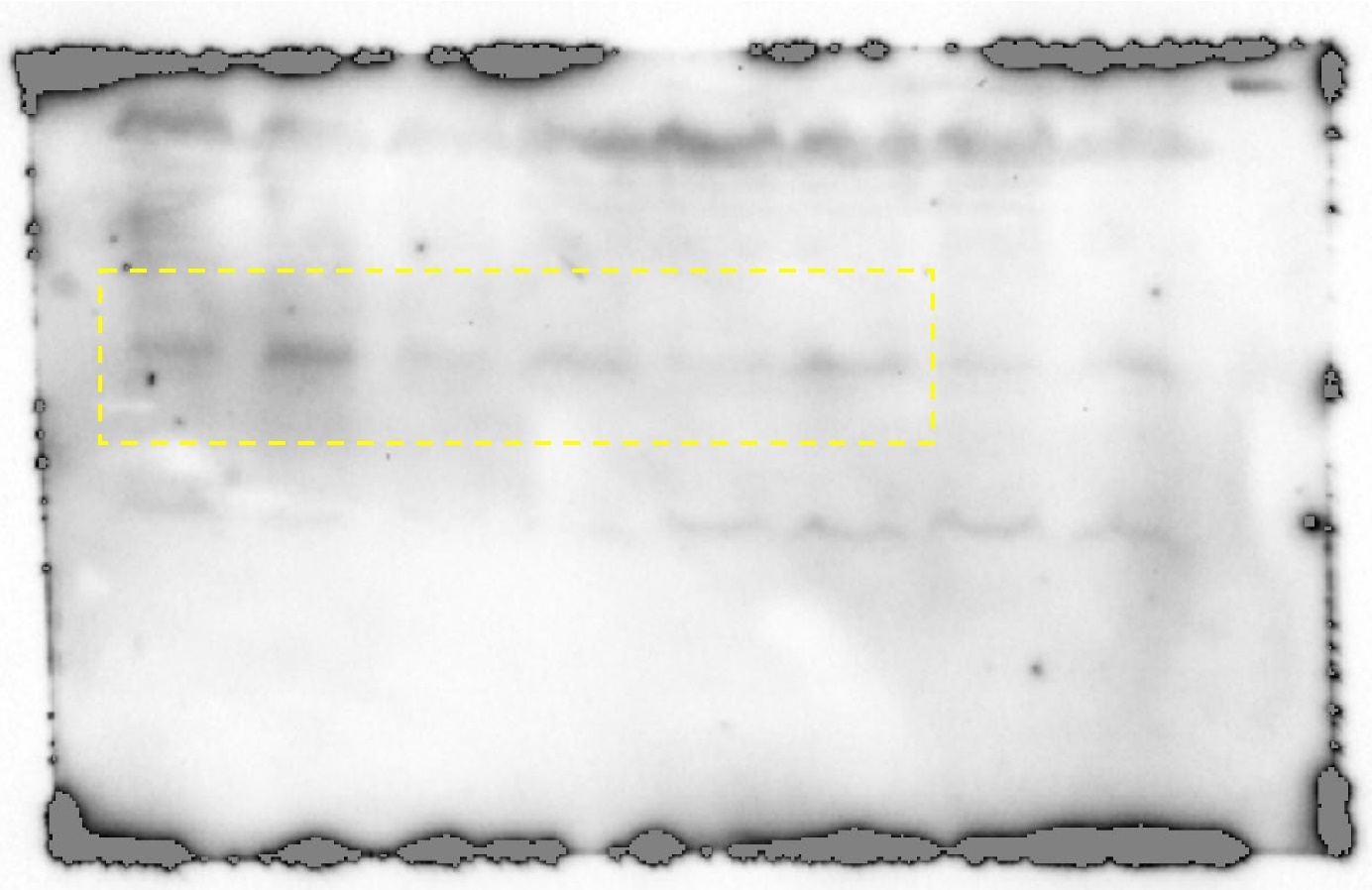


**Fig. S6. Whole membrane images of CCL3 immunoblotting in Fig. 6C**


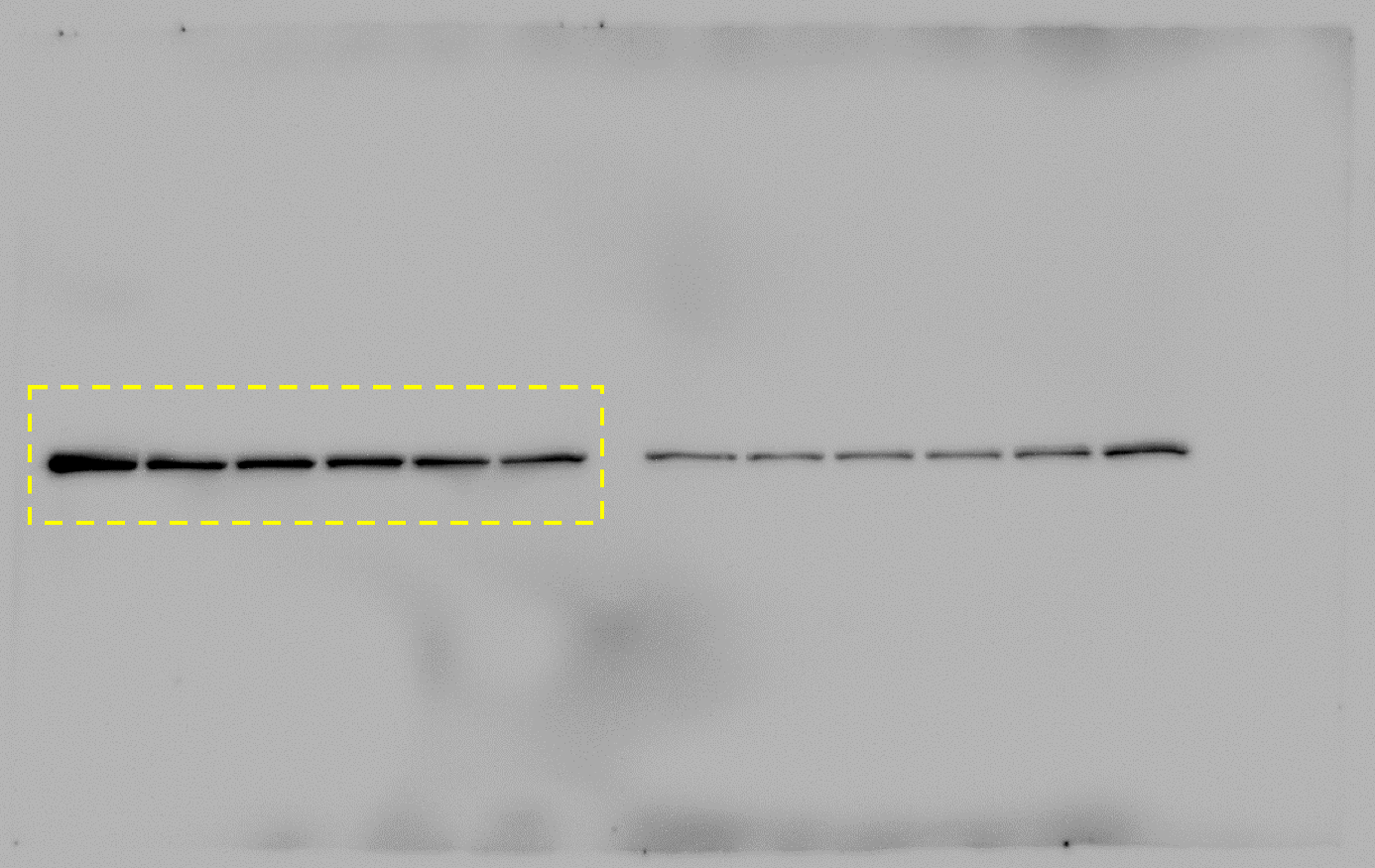


**Fig. S7. Whole membrane images of β-actin immunoblotting in Fig. 6C**

**
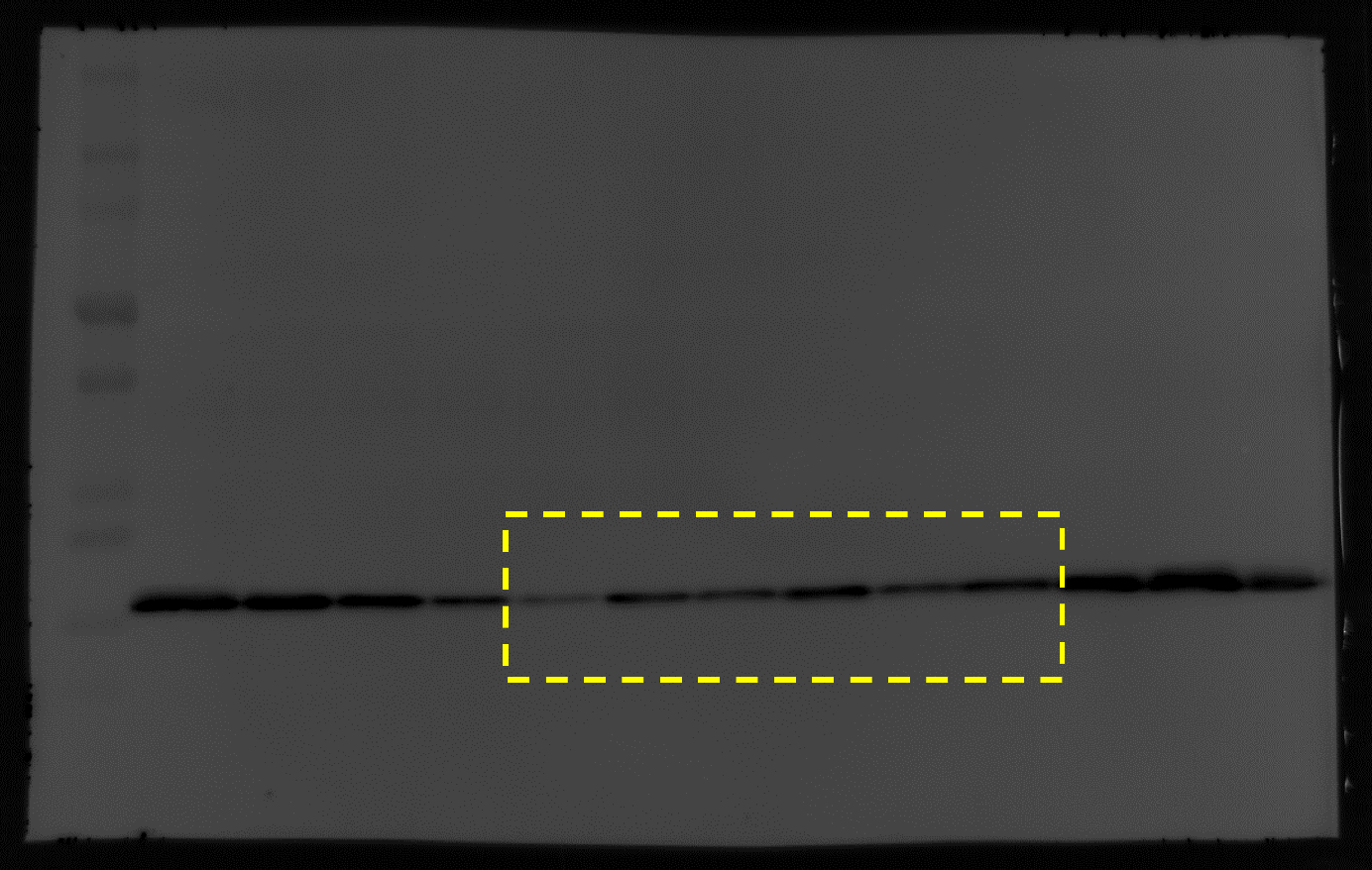
**

**Fig. S8. Whole membrane images of acetyl-histone H3 immunoblotting in Fig. S4B**

**
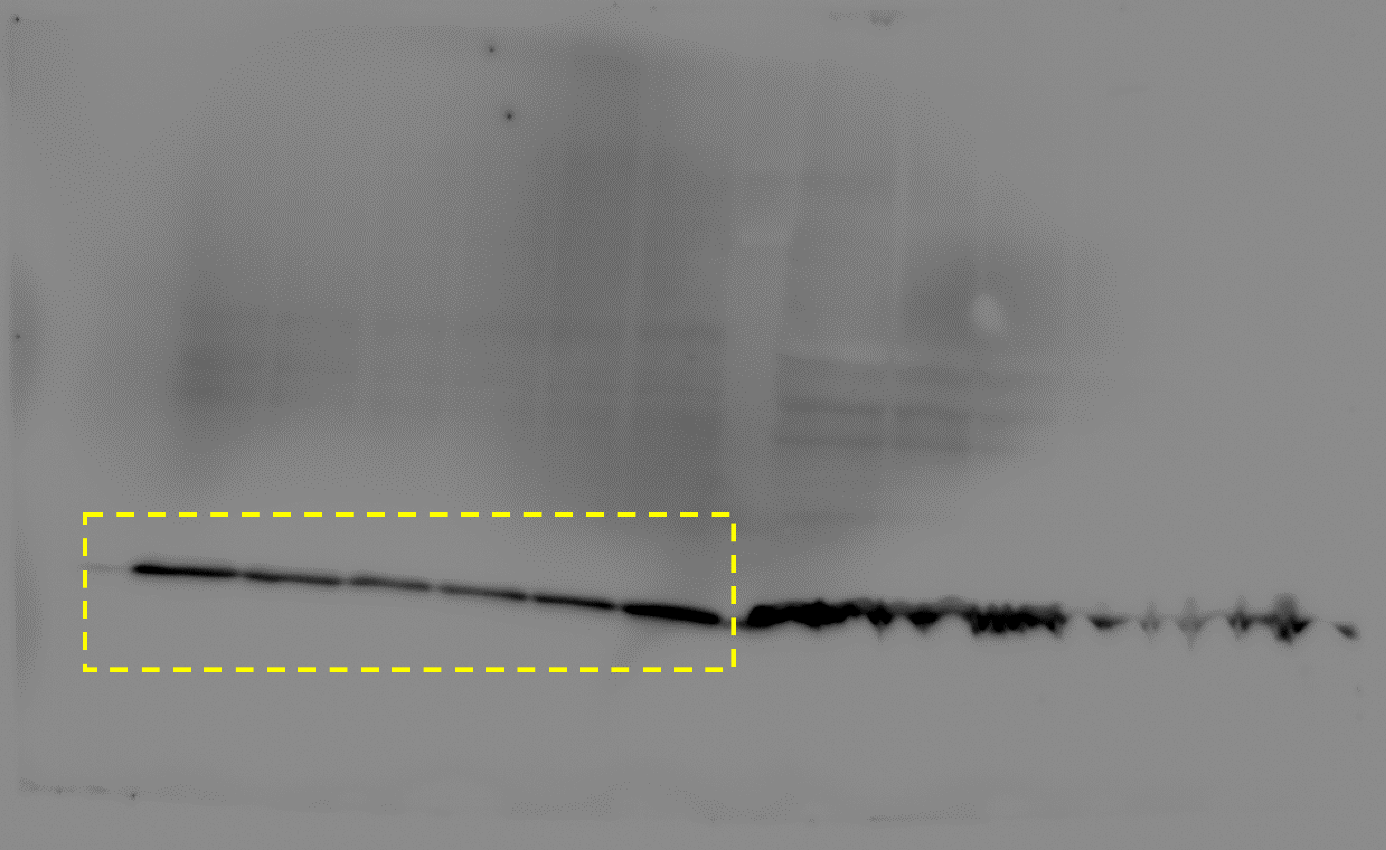
**

**Fig. S9. Whole membrane images of histone H3 immunoblotting in Fig. S4B**
